# A Supramolecular Thermal Switch for Precision Pyroptosis via Host−Guest Recognition and Electrostatic Interactions

**DOI:** 10.1002/advs.76664

**Published:** 2026-07-20

**Authors:** Dan Wu, Jie Zhou, Yibin Cao, Kunmin Ping, Borui Zhao, Yanrong Yang, Chunyang Yu, Xinyang Yu, Shaolong Qi

**Affiliations:** ^1^ State Key Laboratory of Advanced Separation Membrane Materials Zhejiang Key Laboratory of Advanced Polymer Materials Modification and Application Technology College of Materials Science and Engineering Zhejiang University of Technology Hangzhou P. R. China; ^2^ State Key Laboratory of Synergistic Chem‐Bio Synthesis School of Chemistry and Chemical Engineering Shanghai Key Laboratory of Electrical Insulation and Thermal Aging Shanghai Jiao Tong University Shanghai P. R. China; ^3^ Department of Chemistry Ministry of Education Key Laboratory of Bioorganic Phosphorus Chemistry & Chemical Biology Tsinghua University Beijing P. R. China

**Keywords:** Ca^2+^ overload, electrostatic interactions, host–guest complex, pyroptosis, supramolecular chemistry

## Abstract

Cytomembrane‐puncturing gasdermin (GSDM)‐mediated pyroptosis activates antitumor immunity through the release of immunogenic cellular contents and proinflammatory cytokines. However, programmable and precise pyroptosis remains challenging due to the rigidity, inefficiency, and systemic toxicity of conventional inducers. Herein, a supramolecular thermal switch is engineered to enable precision pyroptosis induction. Triiodide ions (I_3_^−^) are stabilized within β‐cyclodextrin (*β‐CD*) via host–guest recognition, enhancing payload efficiency and bioavailability, while calcium ions (Ca^2^^+^), anchored on the periphery of *β‐CD* through electrostatic Ca^2^^+^/carboxylate interactions, nucleate CaCO_3_ crystal growth. The resulting CaCO_3_ matrix acts as a sustained Ca^2+^ reservoir while preventing premature leakage of both Ca^2+^ and I_3_^−^. In the acidic tumor microenvironment, CaCO_3_ decomposes, enabling site‐specific release of I_3_^−^ and Ca^2+^ via a pH‐responsive disassembly mechanism. Under spatiotemporally controlled near‐infrared (NIR) laser irradiation, the boron dipyrromethene (BODIPY)‐based thermal switch is activated, converting biocompatible I_3_^−^ into toxic iodine (I_2_) and triggering a cascade of intracellular oxidative stress, mitochondrial damage and calcium buffer collapse. Synergizing with dysregulated Ca^2^^+^ homeostasis, exogenous Ca^2^^+^ burst induces Ca^2^^+^ overload and cysteine‐aspartic acid protease‐3 (caspase‐3)/GSDME‐mediated pyroptosis, thereby connecting innate and adaptive immunity to inhibit tumor growth and metastasis. This supramolecular engineering strategy presents a promising approach for potentiating cancer immunotherapy.

## Graphical abstract

A supramolecular pyroptotic thermal switch encapsulates I_3_^−^ within β‐cyclodextrin (β‐CD) and undergoes CaCO_3_ mineralization. Upon lysosomal acidification, CaCO_3_ is degraded, releasing Ca^2^^+^ and I_3_^−^. Concurrently, BODIPY activation converts I_3_^−^ into I_2_, inducing oxidative stress and Ca^2^^+^ overload, which together trigger caspase‐3/GSDME‐mediated pyroptosis. This pyroptotic cell death elicits a potent antitumor immune response, effectively inhibiting tumor recurrence and metastasis.

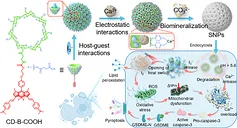

## 1 Introduction

Pyroptosis, a cysteine‐aspartic acid protease‐ (caspase‐) and gasdermin (GSDM) family‐mediated inflammatory programmed cell death, functions as an immunological adjuvant that promotes antigen‐presenting cell maturation and recruitment, eliciting robust immune activation [1−3]. Recent advances reveal that Ca^2+^ overload enables to activate the caspase‐3/GSDME‐dependent pyroptosis pathway, generating a storm of tumor associated antigens to stimulate robust immune responses [4, 5]. While Ca^2+^‐based biomineralizations have been explored for tumor microenvironment‐responsive release, single‐ion therapy is hampered by brief systemic circulation, dose‐dependent systemic toxicity and inefficiency pyroptosis induction [6, 7]. A more fundamental challenge lies in the intelligent calcium buffering systems of tumor cells, which dynamically maintain Ca^2+^ homeostasis via coordinated activity of membrane electrogenic Ca^2+^ channels and subcellular organelles (e.g., mitochondria), thereby resisting Ca^2+^ overload [8, 9]. Molecular iodine (I_2_) can induce oxidative stress and lipid peroxidation through excessive reactive oxygen species (ROS) generation, demonstrating potential to disrupt mitochondrial function [10−12]. Furthermore, I_2_ sublimates at 45°C, functioning as a thermal switch to regulate Ca^2+^ overload [13]. However, its inherent off‐target toxicity severely limits biomedical implementations. A prodrug strategy utilizing triiodide ions (I_3_^−^) as a benign disguise, capable of acid‐ and thermal‐triggered conversion to cytotoxic I_2_, offers a promising solution. A critical hurdle is the inefficient loading of free I_3_^−^ through traditional encapsulation approaches (e.g., covalent modification or physical embedding), accounting for the scarcity of developed delivery systems. The key technological challenge remains establishing a lethal “mitochondrial dysfunction‐Ca^2+^ overload‐pyroptosis” cycle by achieving spatiotemporally controlled corelease of Ca^2+^ and I_3_^−^ within tumor cells. Therefore, developing advanced co‐loading strategies specifically engineered for Ca^2+^ and I_3_^−^ is critical for pyroptosis‐potentiated cancer immunotherapy.

Supramolecular chemistry, which exploits non‐covalent interactions such as hydrogen bonding, *π*–*π* stacking, electrostatic interactions and host–guest recognition, offers a versatile platform for designing novel delivery systems with multifunctional therapeutic encapsulation capabilities [14−20]. The dynamic and reversible nature of these non‐covalent interactions endows supramolecular architectures with stimuli‐responsive behavior and reduced side effects [21−27]. Specifically, FDA‐approved *β*‐cyclodextrin (*β*‐CD) can encapsulate I_3_^−^ in a 1:1 stoichiometry within its hydrophobic cavity, thereby enhancing the physiological stability and bioavailability of I_3_^−^ through supramolecular sequestration [28−31]. Meanwhile, Ca^2+^ can be stabilized in aqueous solution by introducing carboxylate anions (COO^−^) via electrostatic interactions, which prevents its premature leakage and facilitates controlled release within tumor microenvironment (TME) [32−34]. Consequently, leveraging supramolecular chemistry to co‐deliver Ca^2+^ and I_3_^−^ presents a highly promising strategy for pyroptosis therapy indued by Ca^2+^ overload.

**SCHEME 1:**
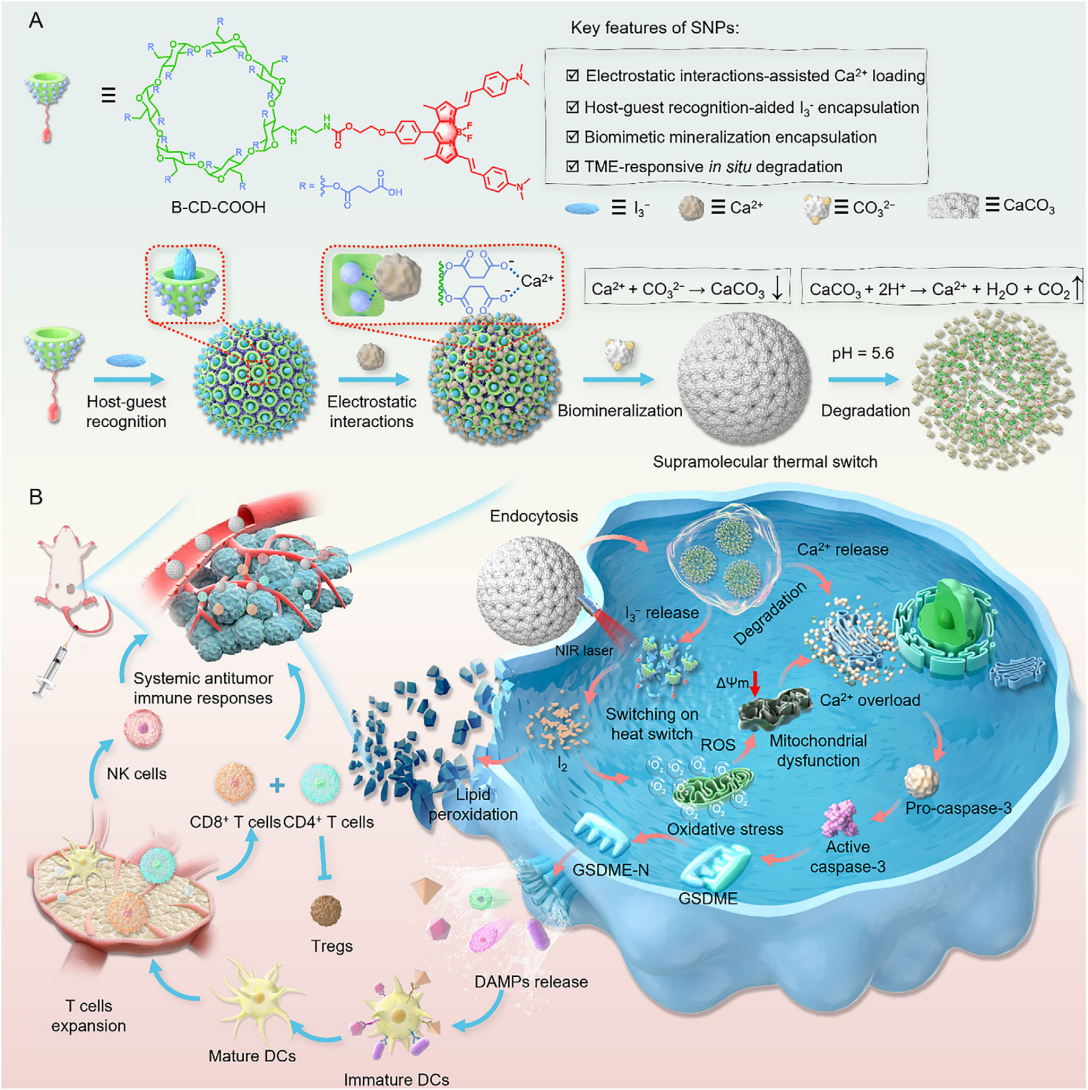
Schematic illustration of a supramolecular thermal switch designed for precision pyroptosis induction and cascade‐amplified cancer therapy. (A) Fabrication process of the supramolecular thermal switch for precision pyroptosis. The system is constructed via host−guest complexation of I_3_^−^ within the cavity of *β*‐CD, followed by Ca^2+^ stabilization on the carboxylate‐functionalized surface and subsequent CaCO_3_ biomineralization. (B) Therapeutic mechanism of supramolecular thermal switch for cascade‐amplified cancer therapy. Following cellular uptake, the lysosomal acidity dissolves the CaCO_3_ matrix, co‐releasing Ca^2+^ and I_3_^−^. Upon NIR irradiation, the BODIPY‐based thermal switch is activated, converting I_3_^−^ into I_2_ and initiating a destructive cascade of oxidative stress, mitochondrial damage and Ca^2+^ dyshomeostasis. The endogenous Ca^2^^+^ dyshomeostasis and exogenous Ca^2^^+^ release synergistically induce Ca^2+^ overload, activating the caspase‐3/GSDME‐mediated pyroptosis. The release of DAMPs promotes dendritic cell maturation and T lymphocyte recruitment, thereby stimulating a potent systemic antitumor immunity to suppress tumor growth and metastasis.

Herein, we develop a supramolecular thermal switch that precisely induces pyroptosis through host–guest recognition and electrostatic interactions to amplify cancer immunotherapy (Scheme 1). The hydrophobic cavity of *β*‐CD host forms a stable host–guest complex with I_3_^−^, significantly enhancing its loading efficiency and bioavailability. The exterior of *β*‐CD is covalently functionalized with a near‐infrared (NIR) photothermal agent and multiple carboxyl groups, the latter of which electrostatically stabilize Ca^2+^. These bound Ca^2+^ ions subsequently serve as templates for CaCO_3_ mineralization, yielding a biomineralized supramolecular architecture, which functions dually as an exogenous Ca^2+^ reservoir and a protective barrier against the premature leakage of unstable I_3_^−^. After endocytosis by tumor cells, the lysosomal acid‐triggered dissolution of the CaCO_3_ core enables rapid co‐release of Ca^2+^ and I_3_^−^. Upon NIR activation, the boron dipyrromethene (BODIPY)‐based photothermal switch drives the decomposition of I_3_^−^ into I_2_, initiating a programmed cascade of oxidative stress, mitochondrial damage and calcium buffering system collapse. The synergistic effect of endogenous Ca^2+^ dyshomeostasis and exogenous Ca^2^^+^ release induces Ca^2+^ overload, specifically activating the caspase‐3/GSDME‐mediated pyroptotic pathway. This process elicits robust immunogenic cell death (ICD), stimulating potent systematic antitumor immune responses that effectively suppress tumor growth and metastasis. Therefore, this hybrid supramolecular pyroptotic switch represents an innovative therapeutic paradigm for potentiating cancer therapy.

## 2 Results

### 2.1 Fabrication and Characterization of SNPs

The macrocyclic host B‐CD‐COOH was synthesized through a multistep route with a high overall yield (Figure 1A and Figures S1–S8). The synthesis commenced with the selective mono‐functionalization of *β*‐CD using tosyl chloride (TsCl), yielding the key intermediate *β*‐CD‐OTs. Subsequent nucleophilic substitution with ethanediamine (EDA) introduced an amine handle, producing *β*‐CD‐NH_2_. To ensure regioselective succinylation, the primary amines were temporarily protected with di‐tert‐butyl dicarbonate (Boc_2_O) before reacting with succinic anhydride. This procedure thus generated HOOC‐CD‐NHBoc, bearing approximately 15 carboxyl groups per *β*‐CD molecule. Finally, the target host HOOC‐CD‐NH_2_ was obtained after efficient removal of the Boc protecting groups using trifluoroacetic acid.

**FIGURE 1:**
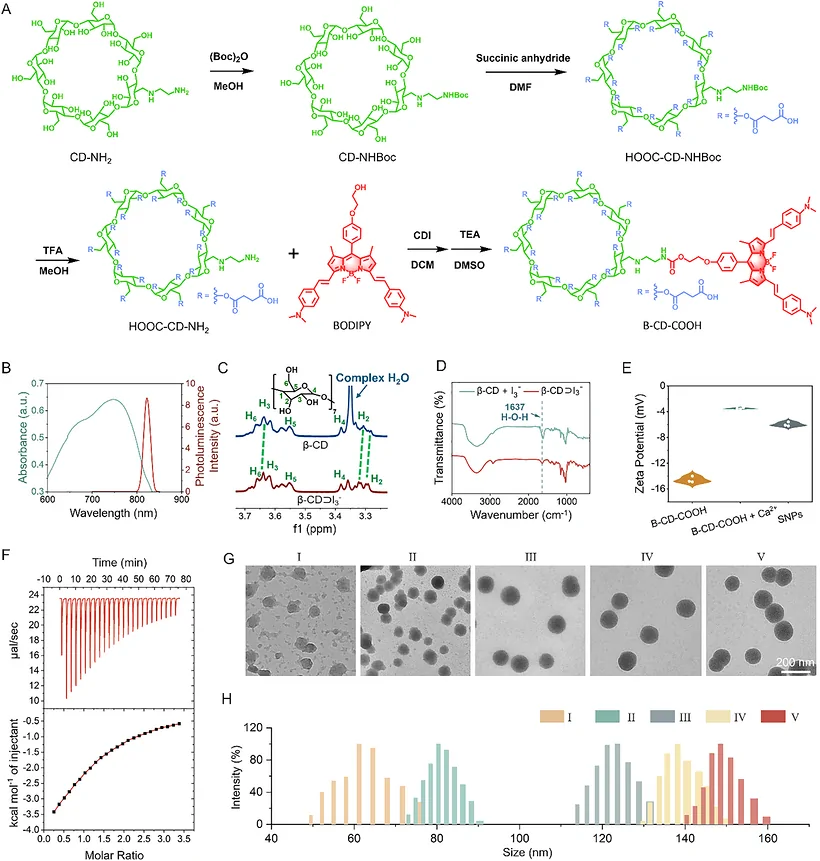
Characterization of the SNPs and their assembly process. (A) Synthetic route of B‐CD‐COOH. (B) UV–vis absorption and fluorescence emission spectra of B‐CD‐COOH. (C) ^1^H NMR spectra (400 MHz, 298 K, DMSO‐*d_6_*) of *β*‐CD (1.00 mM) and the *β*‐CD⊃I_3_^−^ host–guest complex (1.00 mM). (D) FTIR spectra of a physical mixture (*β*‐CD + I_3_^−^) and the host–guest complex (*β*‐CD⊃I_3_^−^). (E) Zeta potential of B‐CD‐COOH, B‐CD‐COOH with Ca^2+^ and the final SNPs. (F) ITC titration profile for the host−guest complexation between *β*‐CD and I_3_^−^. (G) TEM images and (H) hydrodynamic size distributions of nanoparticles prepared under varying biomineralization conditions. I: 6.18 mg mL^−1^ CaCl_2_, 2.95 mg mL^−1^ Na_2_CO_3_, stirred 24 h; II: 6.18 mg mL^−1^ CaCl_2_, 5.90 mg mL^−1^ Na_2_CO_3_, stirred 12 h; III: 6.18 mg mL^−1^ CaCl_2_, 5.90 mg mL^−1^ Na_2_CO_3_, stirred 24 h; IV: 6.18 mg mL^−1^ CaCl_2_, 5.90 mg mL^−1^ Na_2_CO_3_, stirred 48 h; V: 6.18 mg mL^−1^ CaCl_2_, 11.8 mg mL^−1^ Na_2_CO_3_, stirred 24 h.

The NIR‐responsive BODIPY photothermal switch was synthesized in a multi‐step procedure (Figure S9). The process began with the trifluoroacetic acid‐catalyzed condensation of 2,4‐dimethyl pyrrole with 1,4‐hydroxyethoxybenzaldehyde, followed by oxidative aromatization using tetrachloro‐1,4‐benzoquinone to yield a dipyrromethene ligand. Subsequent chelation with borontrifluoride diethyl etherate produced the BODIPY precursor. To extend the π‐conjugation, the BODIPY core was functionalized with dimethylaminobenzaldehyde via a Knoevenagel condensation, yielding the final BODIPY photosensitizer which exhibited a characteristic absorption maximum at 760 nm and a corresponding emission at 822 nm (Figure 1B). Site‐specific conjugation was then achieved through a 1,1′‐carbonyldiimidazole (CDI)‐mediated coupling between the hydroxy of BODIPY and the primary amine of HOOC‐CD‐NH_2_, resulting in a BODIPY‐functionalized cyclodextrin with preserved carboxyl groups (B‐CD‐COOH).

The formation of a stable *β*‐CD⊃I_3_^−^ inclusion complex was first validated by size compatibility analysis. The 𝛽‐CD cavity (maximum inside diameter = 7.80 Å) readily accommodates I_3_^−^ (van der Waals diameter = 4.22 Å) (Figure S10), yielding a favorable size compatibility ratio (cavity diameter/guest diameter) of 1.85. The host–guest complexation was verified by ^1^H NMR spectroscopy. As displayed in Figure 1C, where the inclusion of I_3_^−^ induced significant chemical shift perturbations in the cavity protons and caused the disappearance of the cavity water signal at 3.33 ppm, demonstrating dehydration of the hydrophobic cavity. This was further corroborated by fourier transform infrared (FTIR) analysis, which showed a marked decrease in the bound water bending vibration at 1637 cm^−1^ for the *β*‐CD⊃I_3_^−^ complex compared to a physical mixture (Figure 1D). Isothermal titration calorimetry (ITC) determined a 1:1 binding stoichiometry with an association constant (*K*_a_) of 1.62 × 10^3^ M^−1^ (Figure 1F and Figure S11), confirming the complex is enthalpically driven and stable in aqueous solution. Separately, zeta potential analysis quantified the electrostatic interactions. The potential of B‐CD‐COOH shifted markedly from −14.6 to −3.4 mV upon Ca^2^^+^ addition, demonstrating complexation with carboxylate groups. Subsequently, the introduction of CO_3_^2−^ shifted the potential to ‐6.1 mV (Figure 1E), confirming successful CaCO_3_ biomineralization. This mineralization approach offered exceptional Ca^2+^ retention and minimal I_3_^−^ leakage, ensuring circulatory stability without comprising the therapeutic Ca^2+^ reservoir.

Subsequently, we systematically optimized the biomineralization parameters, specifically the Ca^2+^/CO_3_^2−^ ratio and mineralization time, to obtain monodisperse nanoparticles suitable for intravenous administration. As shown in Figure 1G,H, nanoparticle growth exhibited a concentration dependence. At a CO_3_^2−^ concentration of 2.95 mg mL^−1^, irregular spherical nanoparticles with a constrained average diameter of 62 nm were formed (Figure 1G‐I). Increasing the CO_3_^2−^ concentration to 5.90 mg mL^−1^ produced well‐defined nanoparticles with an average size of 124 nm and a symmetrical Gaussian size distribution (Figure 1G‐III). However, a further increase to 11.8 mg mL^−1^ led to excessive growth (148 nm) and obvious particle fusion (Figure 1G‐V). The influence of churning time was also investigated. Stirring for 12 h yielded non‐uniform nanoparticles (Figure 1G‐II), whereas extending the time to 24 h allowed the biomineralization to reach equilibrium, resulting in well‐dispersed, homogeneous particles with an average diameter of 124 nm (Figure 1G‐III). Prolonged churning for 48 h caused overgrowth, producing hydrated particles with a diameter of 138 nm but with an elevated polydispersity index (PDI) (Figure 1G‐IV and Figure S12). Based on these results, the optimal conditions for generating suitable SNPs were determined to be: 6.18 mg mL^−1^ of CaCl_2_, 5.90 mg mL^−1^ of Na_2_CO_3_, and a churning time of 24 h.

The supramolecular nanoparticles (SNPs) were constructed through a hierarchical assembly process. Initially, I_3_^−^ was encapsulated within the *β*‐CD cavity via host–guest complexation. The periphery of the resulting complex was then electrostatically coordinated with Ca^2+^ ions, which served as templates for subsequent biomineralization. The obtained SNPs possessed a uniform spherical morphology and a hydrodynamic diameter of 124 nm, making them suitable for intravenous injection and potential tumor accumulation via the enhanced permeability and retention (EPR) effect. Comprehensive elemental and structural analyses confirmed the successful integration of all components. Elemental mapping showed a homogenous distribution of N, O, F, Ca and I throughout the SNPs matrix (Figure 2A), which was consistent with energy‐dispersive x‐ray spectroscopy (EDX) results (Figure S13). X‐ray photoelectron spectroscopy (XPS) further confirmed the presence of C (1s), B (1s), O (1s), I (3d) and Ca (2p) (Figure 2B). The Ca 2p spectrum exhibited doublet peaks at 345.2 eV (2p_3/2_) and 348.8 eV (2p_1/2_) (Figure S14), confirming the bivalent state of calcium. Meanwhile, the I 3d spectrum exhibited characteristic doublets at 616.4 eV (3d_5/2_) and 628.0 eV (3d_3/2_) (Figure S14). Finally, x‐ray powder diffraction (XRD) analysis, after subtracting background contributions from potassium iodide (KI) and sodium chloride (NaCl), revealed that all characteristic diffraction peaks of the SNPs could be indexed to the calcite phase of CaCO_3_ (JCPDS no. 05–0586) (Figure 2C), evidencing the presence of cubic CaCO_3_ crystals within the structure.

**FIGURE 2:**
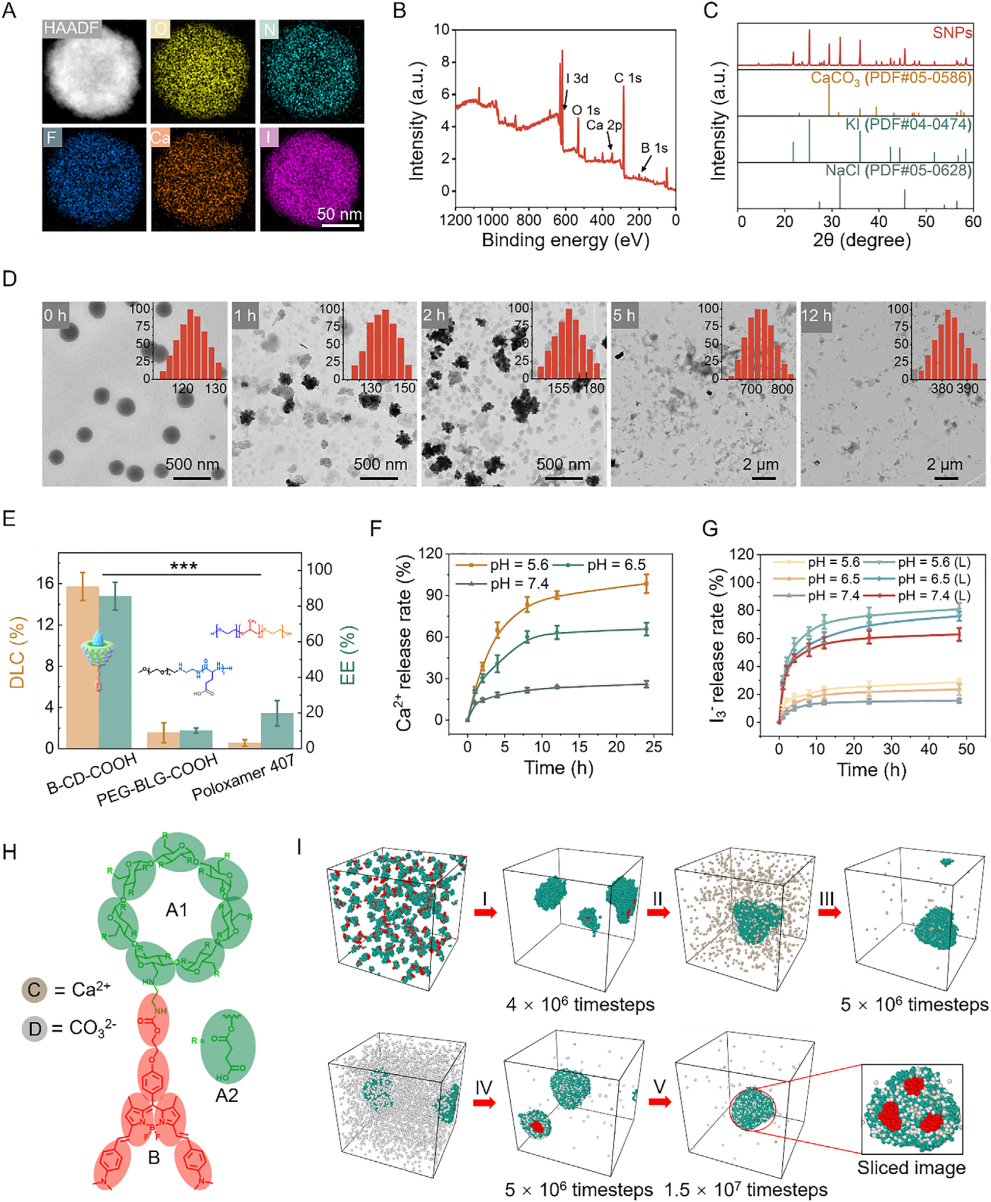
Comprehensive physicochemical characterization and proposed assembly mechanism of the SNPs. (A) Element mapping analysis of the SNPs. (B) XPS spectrum of the SNPs. (C) XRD patterns of the SNPs, with reference patterns for calcite CaCO_3_, KI and NaCl. (D) Time‐dependent structural disintegration of SNPs in an acidic environment (pH 5.6), as visualized by TEM images and quantified by the evolution of DLS over 12 h. (E) Comparative analysis of DLC and EE for I_3_^−^ across different carries. Cumulative release profiles of (F) Ca^2+^ and (G) I_3_^−^ from SNPs under different simulated physiological conditions. (H) Molecular structures of the key building blocks: B‐CD‐COOH, Ca^2+^ and CO_3_^2−^. (I) Proposed time‐resolved self‐assembly mechanism: (I) formation of the core B‐CD‐COOH complex, (II, III) electrostatic coordination of Ca^2+^ ions with carboxyl groups, (IV, V) oriented growth of a CaCO_3_ mineral shell via reaction with CO_3_^2−^, leading to the final SNPs. Data in (E‐G) are presented as mean ± SD (n ≥ 3). Statistical significance was calculated via ordinary one‐way ANOVA with a Tukey's test. ^*^ *p* < 0.05; ^**^ *p* < 0.01; ^***^ *p* < 0.001; ^****^ *p* < 0.0001.

The SNPs exhibited exceptional colloidal stability under physiological conditions, maintaining consistent hydrodynamic diameters after 24 h of incubation in H_2_O, PBS, RPMI 1640 and 10% FBS (Figure S15). In contrast, exposure to acidic buffer triggered a rapid structural disassembly, as evidenced by a progressive increase in particle size and the development of irregular morphologies (Figure 2D). After 5 h of incubation in acid, extensive micron‐scale precipitation formed, indicative of matrix cross‐linking during the initial phase of dissolution. Extending the incubation to 12 h resulted in the near‐complete disappearance of high‐contrast mineral residues, confirming the thorough degradation of the CaCO_3_ framework. These results validated that the SNPs possess the desired dual functionality, offering high stability during systemic circulation to reduce off‐target toxicity and undergoing selective, rapid disintegration within acidic tumor microenvironment to activate the therapeutic payload.

### 2.2 The Critical Role of Noncovalent Interactions in Enhancing Ca^2+^ and I_3_^−^ Loading

Ultraviolet (UV) spectrophotometry was utilized to quantify the I_3_^−^ loading capacity of B‐CD‐COOH. Compared to PEG‐BLG‐COOH and poloxamer 407 polymers which lacked *β*‐CD modification, B‐CD‐COOH exhibited an 8.4‐fold and a 4.3‐fold higher encapsulation efficiency (EE), respectively. Moreover, B‐CD‐COOH showed significantly higher drug loading contents (DLC) for I_3_^−^ (Figure 2E and Figure S16), highlighting the essential role of host–guest recognition in achieving highly efficient I_3_^−^ encapsulation.

To achieve spatiotemporally controlled I_2_ release by leveraging the thermal decomposition of I_3_^−^, a photothermally switch was engineered into the supramolecular architecture. Upon NIR irradiation, the SNPs solution showed an immediate color transition from yellowish‐brown to green (Figures S17 and S18), providing direct visual evidence of the I_3_^−^‐to‐I_2_ transformation and subsequent I_2_ sublimation. The release kinetics of Ca^2+^ and I_2_ were systematically monitored under different conditions. As shown in Figure 2F, merely 26.0% of encapsulated Ca^2+^ was released at physiological pH (7.4), certifying the exceptional stability of SNPs in circulation. In stark contrast, a near‐complete Ca^2+^ release (98.0%) was achieved at a weakly acidic pH (5.6), disclosing a pronounced pH‐responsive release characteristic through acid‐triggered dissolution of the CaCO_3_ matrix. While the acidic condition (pH 5.6) alone enhanced I_2_ release, the maximum release rate remained below 25%, suggesting that host–guest confinement within the *β*‐CD cavity effectively prevented premature release and potential off‐target toxicity. Remarkably, upon NIR irradiation, the I_2_ release efficiency significantly increased to 81.0% (Figure 2G), demonstrating that photothermal activation was the dominant trigger for I_3_^−^ thermolysis and consequent I_2_ liberation. These inspiring results affirmed that our supramolecular hierarchical assembly successfully integrates spatiotemporally controlled activation and tumor microenvironment responsiveness to achieve precise pyroptosis induction.

Dissipative particle dynamics (DPD), a particle‐based mesoscale simulation method, was employed to investigate the self‐assembly mechanism of the SNPs. In this framework, atoms or molecular groups are coarse‐grained into interactive beads, whose dynamics are governed by Newton's equations of motion via pairwise conservative, dissipative, and random forces. In our model, the B‐CD‐COOH molecule was represented as a hydrophilic domain (A1 + A2, 21 beads) and a hydrophobic core (B, 6 beads), while Ca^2+^ and CO_3_^2−^ were modeled as distinct “C” and “D” beads, respectively (Figure 2H). Time‐resolved snapshots in Figure 2I captured the dynamic self‐assembly pathway of SNPs, revealing their morphological transitions. Following an initial homogeneous dispersion, B‐CD‐COOH rapidly self‐assembled into micellar aggregates through cooperative H‐bonds and *π*–*π* stacking. The micellar intermediates underwent hierarchical assembly into larger multi‐micellar aggregates through oriented attachment by 1.5 × 10^7^ timesteps. Subsequently, Ca^2+^ ions migrated into micellar cores through electrostatically guided permeation. The final stabilization of the system was achieved by CO_3_^2−^‐triggered biomineralization, as clearly visualized in the cross‐sectional view.

### 2.3 Supramolecular Cascade Feedback Loop on Mitochondrial Damage

The intracellular trafficking of SNPs was first monitored by confocal laser scanning microscopy (CLSM). Images showed efficient cellular internalization of the red‐fluorescent SNPs, which exhibited strong colocalization with green Lyso‐Tracker signals (Figure 3A), confirming lysosomal trafficking as the primary internalization pathway. This was further quantified by flow cytometry, which revealed a time‐dependent cellular uptake with a 10‐fold increase in fluorescence intensity from 1 to 8 h post‐incubation (Figure 3B). At the 8 h time point, clear separation between the green lysosomal signal and the red nanoparticle signal (Figures S19 and S20), indicating successful lysosomal escape, where the acidic lysosomal environment degraded the CaCO_3_ matrix and triggered I_2_ release. Endocytosis inhibition assays revealed that SNPs uptake was primarily mediated by clathrin‐ and macropinocytosis‐dependent pathways (Figures S21 and S22). As a well‐established “mitocan”, I_2_ is a potent inducer of cellular oxidative stress. Next, intracellular ROS levels were measured using the redox‐sensitive probe 2,7‐dichlorofluorescin diacetate (DCFH‐DA). As shown in Figure 3C and Figure S23, the SNPs (L)‐treated group generated significantly higher ROS levels compared to all four control groups, confirming the superior capability of our system to induce oxidative stress. ROS overproduction triggers mitochondrial oxidative damage, resulting in mitochondrial dysfunction. Then, mitochondrial membrane potential (MMP) was assessed using JC‐1 staining, where J‐aggregates (red fluorescence) indicates high MMP and monomers (green fluorescence) reflect depolarization. As displayed in Figure 3J, PBS and B‐CD@Ca groups exhibited predominant JC‐1 aggregation, confirming maintained MMP. However, SNPs (L) treatment induced a significant shift to JC‐1 monomers, demonstrating severe mitochondrial depolarization.

**FIGURE 3:**
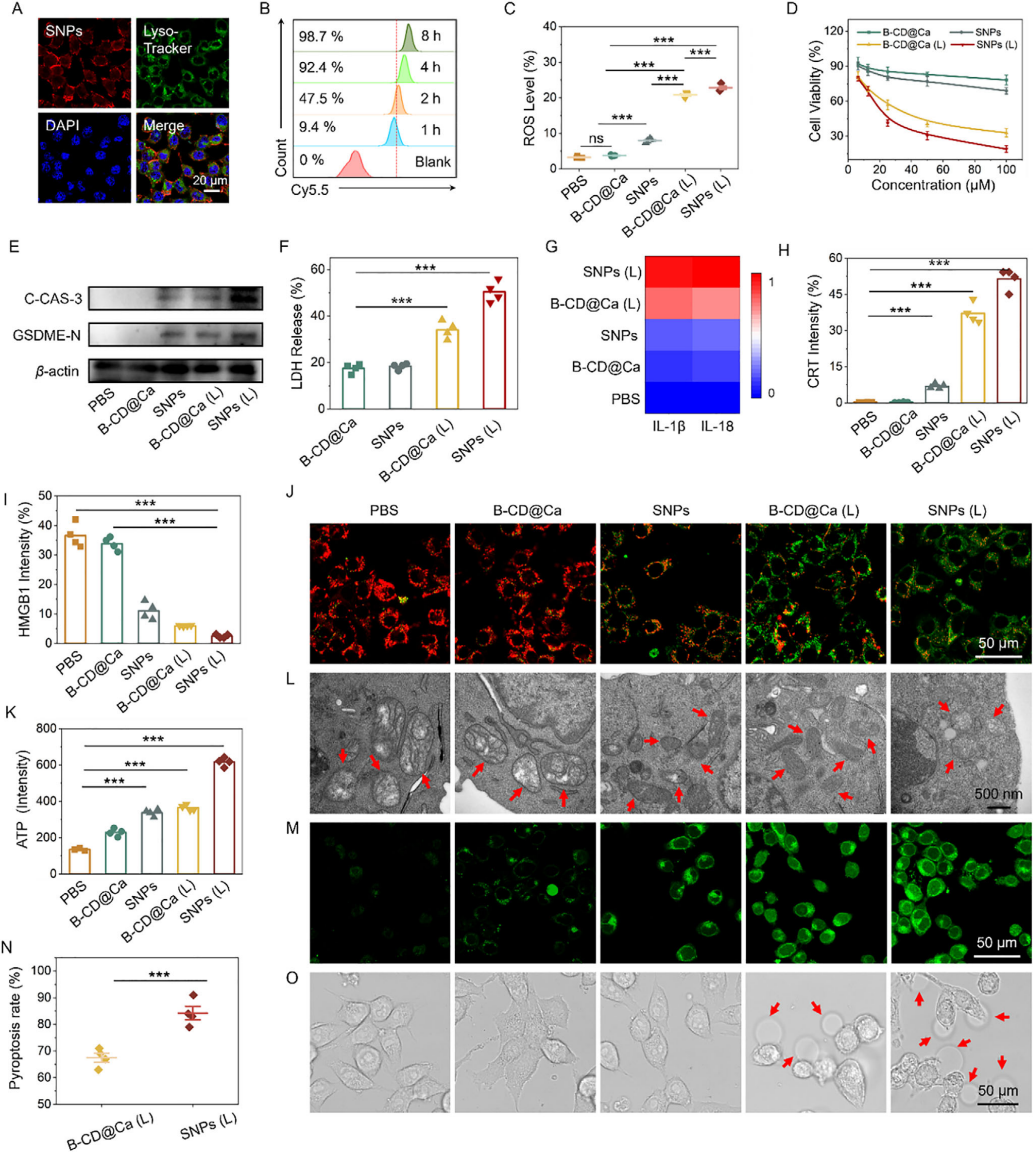
The SNPs initiate a destructive cascade of oxidative stress, mitochondrial damage and calcium buffering system collapse, leading to specific Ca^2+^ overload and ultimately culminating in caspase‐3/GSDME‐mediated pyroptosis and potent ICD. (A) CLSM images of 4T1 cells incubated with SNPs for 2 h, with lysosomal location (Lyso‐Tracker) and nuclear staining (DAPI). (B) Time‐dependent cellular uptake of SNPs quantified by flow cytometry. (C) Cellular ROS levels after different treatments. (D) Cell viability of 4T1 cells post‐treatments. (E) WB analysis of GSDME‐N and C‐CAS‐3. (F) Quantification of LDH release into cell culture supernatants. (G) Secretion levels of IL‐18 and IL‐1𝛽 in supernatants. Quantification of (H) CRT exposure and (I) HMGB1 efflux. (J) CLSM images of JC‐1‐stained 4T1 cells following different treatments. (K) Quantification of ATP release into cell culture supernatants. (L) Bio‐TEM images of mitochondrial ultrastructure. (M) Intracellular Ca^2+^ levels detected by Fluo‐4 AM staining. (N) Pyroptosis frequency across treatment groups. (o) Bright‐field and CLSM micrographs of 4T1 cells post‐treatments. Data are presented as mean ± SD (*n* ≥ 3). Statistical significance was calculated via ordinary one‐way ANOVA with a Tukey's test. ^*^ *p* < 0.05; ^**^ *p* < 0.01; ^***^ *p* < 0.001; ^****^ *p* < 0.0001.

Mitochondrial ultrastructure was then examined by biological transmission electron microscopy (Bio‐TEM). As depicted in Figure 3L, control groups (PBS and B‐CD@Ca) exhibited normal mitochondrial morphology with distinct outer/inner membranes, lamellar cristae and homogeneous matrix granules. Whereas, SNPs (L) treatment induced hallmark mitochondrial pathologies including chromatin condensation, swelling, cavitation and mitochondrial number reduction, demonstrating profound structural disruption. Mitochondria play a central role in regulating Ca^2+^ signaling and maintaining calcium homeostasis [35]. Due to its inability to damage mitochondrial, B‐CD@Ca treatment failed to induce significant Ca^2+^ overload (Figure 3M and Figure S24). In comparison, SNPs and B‐CD@Ca (L) treatments induced moderate calcium overload through either the I_2_‐mediated oxidative stress‐mitochondrial damage‐Ca^2+^ overload cascade or acid‐triggered release of exogenous Ca^2+^ release in the tumor microenvironment. Notably, SNPs (L) generated the most pronounced Ca^2+^ overload, attributable to its supramolecular cascade feedback loop that acts to amplify mitochondrial damage.

Sustained oxidative stress from local ROS accumulation induces significant lipid peroxide (LPO), thereby compromising cell membrane integrity. Quantification using the Liperfluo fluorescent probe revealed that the SNPs (L) group exhibited strong green fluorescence, corresponding to a 5.8‐fold higher LPO level than the PBS group (Figures S25 and S26), thereby confirming severe oxidative stress in treated cells. 3‐(4′,5′‐dimethylthiazol‐2′‐yl)‐2,5‐diphenyltetrazoliumbromide (MTT) assay was performed to assess the in vitro anticancer efficacy of SNPs (L). As depicted in Figure 3D, SNPs (L) showed concentration‐dependent cytotoxicity with a half‐maximal inhibitory concentration (IC_50_) of 22.2 µg/mL, much lower than that of B‐CD@Ca (386.5 µg/mL), SNPs (238.0 µg/mL) and B‐CD@Ca (L) (36.0 µg/mL). In addition, dual Calcein‐AM/PI staining visualized this effect. While B‐CD@Ca and SNPs caused moderate cell death, SNPs (L) treatment resulted in extensive lethality, as evidenced by prominent red fluorescence (Figure S27).

### 2.4 Caspase‐3/GSDME‐Mediated Pyroptosis Signaling Pathway

Next, we sought to elucidate the underlying cell death mechanism and its downstream effects. As shown in Figure 3O, B‐CD@Ca‐ and SNPs‐treated cells maintained a normal morphology similar to the PBS control. In contrast, both the B‐CD@Ca (L) and SNPs (L) groups exhibited distinct pyroptotic hallmarks, including cellular swelling and large bubble‐like protrusions. Notably, plasma membrane integrity was largely compromised in SNPs (L)‐treated cells, implying substantial cytoplasmic content leakage. Consistent with these observations, the pyroptosis incidence was most pronounced in the SNPs (L)‐treated group (Figure 3N). In addition, classic apoptotic features such as nuclear chromatin condensation and fragmentation, cellular shrinkage and apoptotic body formation were minimally observed in SNPs (L) group, proving the selective induction of pyroptosis over apotosis. Moreover, flow cytometric analysis with Annexin V‐FITC/PI double staining confirmed that SNPs (L) induced the highest level of programmed cell death (64.8%), significantly exceeding the rates in the B‐CD@Ca (2.4%), SNPs (37.8%) and B‐CD@Ca (L) (48.7%) groups (Figure S28).

Building on emerging evidence that Ca^2+^ overload activates caspase‐3/GSDME‐mediated pyroptosis, we analyzed key protein markers by Western blotting (WB). SNPs (L) treatment significantly upregulated the expression of both cleaved caspase‐3 (C‐CAS‐3) and the N‐terminal fragment of GSDME (GSDME‐N) (Figure 3E), demonstrating the activation of this specific pyroptosis pathway. The oligomerized GSDME‐N domains form plasma membrane pores, ultimately causing osmotic lysis and the release of intracellular contents. We subsequently quantified characteristic biomarkers, including lactate dehydrogenase (LDH), interleukin‐1*β* (IL‐1*β*) and interleukin‐18 (IL‐18). As shown in Figure 3F,G, SNPs (L) treatment induced significant LDH release and remarkably elevated levels of IL‐1*β* (5.23‐fold) and IL‐18 (4.92‐fold) compared to the PBS control, certifying the induction of robust pyroptotic cell death accompanied by pro‐inflammatory cytokine release.

Pyroptosis, as pro‐inflammatory programmed cell death modality, potently induces ICD through substantial release of damage‐associated molecular patterns (DAMPs) release such as calreticulin (CRT), high mobility group box 1 (HMGB1) and adenosine triphosphate (ATP). CRT serves as an “eat me” signal by binding to phagocytic receptors, thereby promoting the engulfment of dying cells. As depicted in Figure 3H and Figure S29, SNPs (L) treatment induced the most intense CRT‐associated green fluorescence on the cell membrane, significantly exceeding the minimal signals in the PBS, B‐CD@Ca and SNPs control groups. HMGB1, a nuclear architectural protein, translates from the nucleus region to the extracellular matrix during pyroptosis, where it orchestrates innate immune responses by stimulating proinflammatory cytokine production and neutrophil recruitment. In contrast to the predominant nuclear localization observed in PBS and B‐CD@Ca controls, SNPs (L) treatment induced substantial HMGB1 translocation into the extracellular milieu (Figure 3I and Figure S30), a consequence of pyroptosis‐executing pore formation and membrane rupture. ATP released during pyroptosis promotes phagocytic clearance and NLRP3 inflammasome activation, critical for dendritic cells (DCs) recruitment and T cells priming. ATP quantification revealed SNPs (L) induced maximal efflux, representing a 4.69‐fold elevation over PBS (Figure 3K). These results established SNPs (L) as a potent ICD inducer that converts immunologically “cold” tumors into “hot” microenvironments receptive to immunotherapy. To confirm that the observed cell death proceeds specifically through the caspase‐3/GSDME pyroptosis pathway, we performed inhibition experiments using the caspase‐3 inhibitor Z‐DEVD‐FMK. As shown in Figure S31, addition of Z‐DEVD‐FMK effectively abolished the characteristic pyroptotic morphology, including cell swelling and plasma membrane pore formation. Moreover, inhibitor treatment blocked CRT exposure on the cell surface and prevented the nuclear translocation of HMGB1. These results demonstrate that the supramolecular nanoswitch specifically activates the caspase‐3/GSDME‐dependent pyroptosis pathway.

### 2.5 Evaluation of Antitumor Efficacy in Vivo

Prior to in vivo studies, hemolytic assessment confirmed the blood compatibility of the SNPs, which induced negligible hemolysis (<6%) across all tested concentrations (Figure S32). We then evaluated the pharmacokinetic profile of the SNPs in vivo by quantifying BODIPY‐derived UV–vis absorption in serial blood samples. SNPs exhibited superior pharmacokinetic profiles compared to B‐CD‐COOH, with a longer peripheral elimination half‐life (*t*_β1/2_, 13.9 vs. 8.9 h) and central half‐life (*t*_α1/2_, 0.96 h vs. 0.88 h) (Figure 4A). At 24 h post‐injection, 11.7% of the injected dose per gram (ID g^−1^) of the SNPs remained in circulation, whereas B‐CD‐COOH was completely cleared. The area under the concentration‐time curve (AUC) for the SNPs was 3.84‐fold greater than that of B‐CD‐COOH, demonstrating significantly enhanced systemic exposure. To contextualize these results, we further compared the SNPs with two conventional pyroptosis inducers, paclitaxel (PTX) and tetraphenylporphinesulfonate (TPPS). As shown in Figure S33, in contrast to SNPs (central 0.96 h, peripheral 13.9 h), TPPS (0.45 and 32.8 h) and PTX (0.29 and 4.68 h) exhibited markedly poorer pharmacokinetic profiles, underscoring the pharmacokinetic advantage afforded by supramolecular assembly.

**FIGURE 4:**
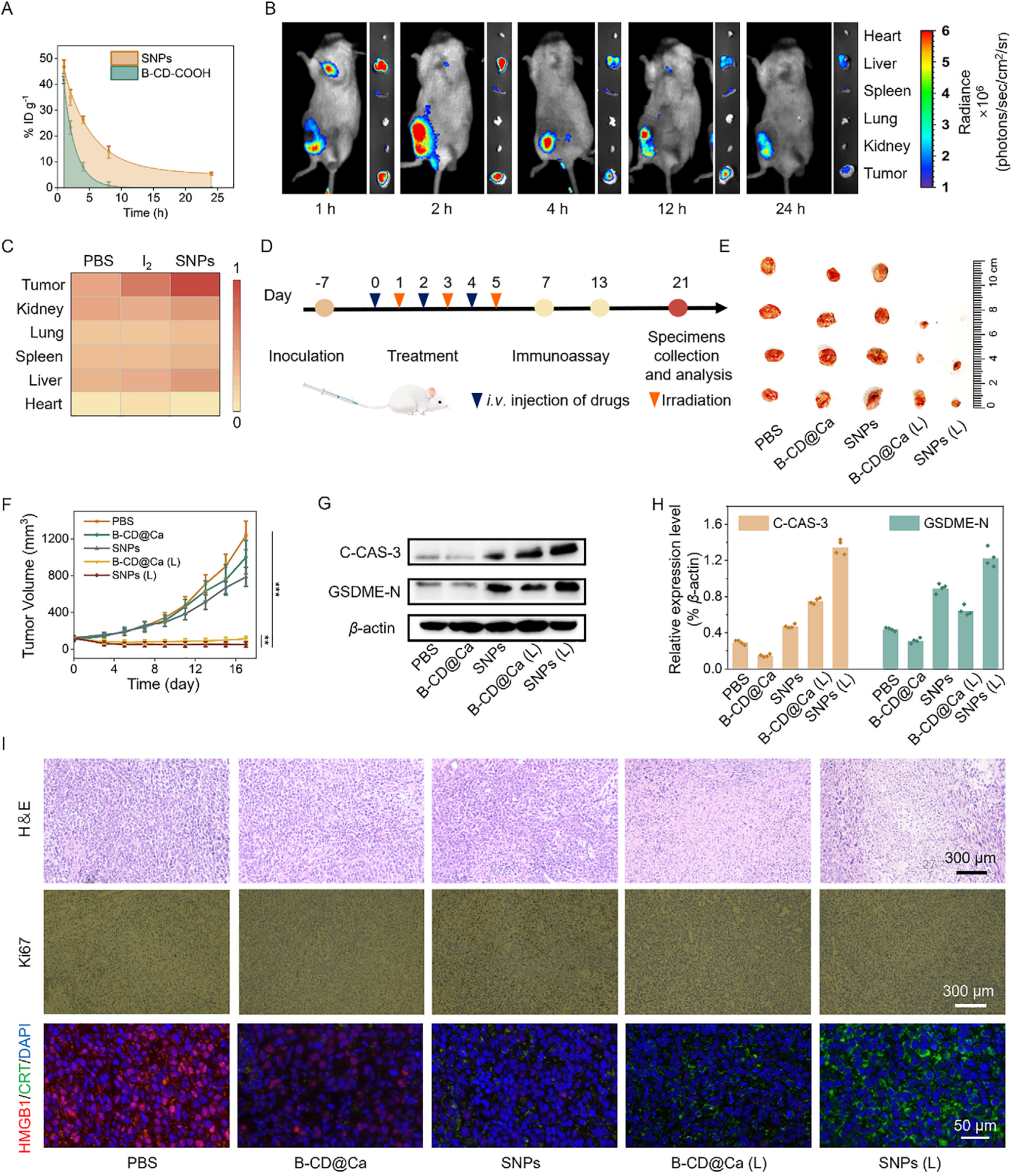
In vivo pharmacokinetics, antitumor efficacy, and mechanism validation of the SNPs (L). (A) Pharmacokinetic profiles of B‐CD‐COOH and SNPs, as determined by monitoring BODIPY‐derived UV–vis absorption spectroscopy over time. (B) In vivo fluorescence whole‐body imaging and corresponding ex vivo organ biodistribution at 1, 2, 4, 12, and 24 h post‐injection. (C) The tissue distribution of I_2_ characterized by UV–vis absorption spectroscopy. (D) Therapeutic regimen for primary tumor therapy. (E) Representative images of excised tumors at the study endpoint. (F) Tumor growth curves across different treatment groups. (G) WB analysis of GSDME‐N and C‐CAS‐3 expression in tumor tissues following different treatments. (H) Quantitative analysis of the protein levels shown in (G). (I) H&E, Ki67, CRT and HMGB1 staining of tumor tissues following different treatments. Data are presented as mean ± SD (*n* ≥ 3). Statistical significance was calculated via ordinary one‐way ANOVA with a Tukey's test. ^*^ *p* < 0.05; ^**^ *p* < 0.01; ^***^ *p* < 0.001; ^****^ *p* < 0.0001.

Furthermore, in vivo fluorescence imaging was used to track the real‐time biodistribution of the SNPs. As shown in Figure 4B, signal was detected in the tumor as early as 1 h post‐injection, peaking at 2 h and remaining clearly visible at 24 h, indicating effective tumor targeting and retention. Ex vivo organ imaging also certified the superior tumor‐specific accumulation of the SNPs, with tumors exhibiting higher fluorescence intensity and more homogeneous distribution compared to major organs (Figure 4B). This pattern was corroborated by direct quantification of I_2_ in tissues, which revealed significantly higher I_2_ level in tumors than in major organs (Figure 4C). Importantly, fluorescence imaging detected no significant nanoparticle accumulation in the thyroid (Figure S34), suggesting that the supramolecular system avoids unwanted iodine deposition and minimizes endocrine disruption risk. Together, these data validated that the hierarchical supramolecular design effectively addresses the delivery challenges associated with systemic I_2_ administration, enabling selective tumor targeting and minimizing off‐target exposure. As a result, pyroptosis induction was spatially precise and confined to tumor sites upon activation of the supramolecular thermal switch.

The in vivo antitumor efficacy of SNPs (L) was assessed in BALB/c mice bearing 4T1 mammary carcinoma. The corresponding treatment regimen was depicted in Figure 4D. 4T1 tumor bearing mice were randomly divided into five groups: (I) PBS, (II) B‐CD@Ca, (III) SNPs, (IV) B‐CD@Ca (L) and (V) SNPs (L). Mice were intravenously injected with assigned nanoformulations at 48 h intervals over three treatment cycles. By the treatment endpoint, the PBS group exhibited a mean tumor volume of 1237 mm^3^, while the SNPs (L) group showed a dramatically reduced volume of 51.3 mm^3^, highlighting its robust antitumor effect (Figure 4E,F). Consistently, the SNPs (L) group exhibited the lowest average tumor weight among all treatment groups (Figure S35). The tumor inhibition rates for B‐CD@Ca, SNPs, and B‐CD@Ca (L) groups were 19.2%, 35.4%, and 90.8%, respectively, all significantly lower than the 96.8% inhibition rate observed in the SNPs (L) group. Histological analysis via H&E staining disclosed extensive nuclear shrinkage and disappearance in the SNPs (L) group, along with the minimal proliferative activity as indicated by Ki67 staining (Figure 4I). Furthermore, WB analysis confirmed SNPs (L) treatment remarkably increased the expressions of pyroptosis‐associated proteins GSDME‐N and C‐CAS‐3 (Figure 4G,H), proving the activation of pyroptosis as the underlying antitumor mechanism.

In parallel, systemic toxicity evaluations underscored the favorable biosafety of the SNPs. Acute toxicity studies at 12 and 60 mg/kg showed no significant changes in body weight (Figure S36), hematological parameters (Figure S37), liver/kidney function (Figure S38), or organ histology (Figure S39). Long‐term toxicity assessment at the therapeutic dose of 12 mg/kg likewise revealed no notable hematological (Figure S40) or histopathological changes (Figure S41). Immunotoxicity was evaluated by monitoring body temperature and histamine level following SNPs administration. Neither body temperature nor histamine level showed significant variations at 6 h post‐injection, and histamine levels remained stable over the 72 h monitoring period (Figures S42 and S43), indicating minimal immunotoxicity. Histological analysis further revealed no evidence of thyroid atrophy, structural disruption, fibrosis, or inflammatory infiltration after repeated administration, indicating that the SNPs do not cause thyroid damage or endocrine disruption (Figure S44). Collectively, these data confirmed that the SNPs induced robust pyroptosis‐driven antitumor effects without detectable acute or chronic systemic toxicity, immunotoxicity or thyroid toxicity, highlighting the translational promise of this supramolecular thermal switch for precise and potent cancer therapy.

### 2.6 Activation of Adaptive and Innate Antitumor Immune Responses

Building upon the promising antitumor activity of SNPs (L), we further investigated its ability to induce ICD, DCs maturation and lymphocyte infiltration. Immunofluorescent analysis showed pronounced CRT translocation and HMGB1 release in SNPs (L)‐treated tumors (Figure 4I), implying robust ICD induction in vivo. As professional antigen‐presenting cells, DCs play a central role in tumor immunosurveillance by engulfing, processing, and presenting tumor‐associated antigens to T lymphocytes. Then, DCs maturation in tumor‐draining lymph nodes (TDLNs) was assessed by flow cytometry. As shown in Figure 5A,B, SNPs (L) treatment increased the proportion of CD80^+^CD86^+^ mature DCs by 11.8‐fold compared to the PBS group, indicating enhanced tumor immunogenicity through pyroptosis‐induced DCs maturation. Mature DCs effectively prime naïve T cells, playing important roles in immune activation and regulation. We further quantified T cell activation markers (CD3, CD4, CD8) using flow cytometry. As shown in Figure 5C–F, the SNPs (L) group exhibited the highest levels of infiltrated T helper cells (CD3^+^CD4^+^, 15.6%) and cytotoxic T lymphocytes (CTLs, CD3^+^CD8^+^, 11.5%) compared to other four groups. Immunofluorescence images similarly demonstrated enhanced T cell infiltration into tumors (Figures S45–S47). By recruiting and expanding immune cells, proinflammatory cytokines critically promote systematic antitumor immunity. We next performed enzyme linked immunosorbent assay (ELISA) to measure serum levels of proinflammatory cytokines including interleukin‐6 (IL‐6), tumor necrosis factor‐α (TNF‐α) and interferon‐γ (IFN‐γ). As shown in Figure 5I, significantly elevated serum levels of IL‐6, TNF‐α and IFN‐γ in the SNPs (L) group, demonstrating systemic antitumor activation. We further compared the antitumor immune responses of SNPs (L) with those induced by PTX and TPPS. Both reference agents elicited attenuated cytokine responses (Figure S48), likely due to their suboptimal delivery efficiency, further confirming the superior capacity of SNPs (L) to trigger immunogenic cell death.

**FIGURE 5:**
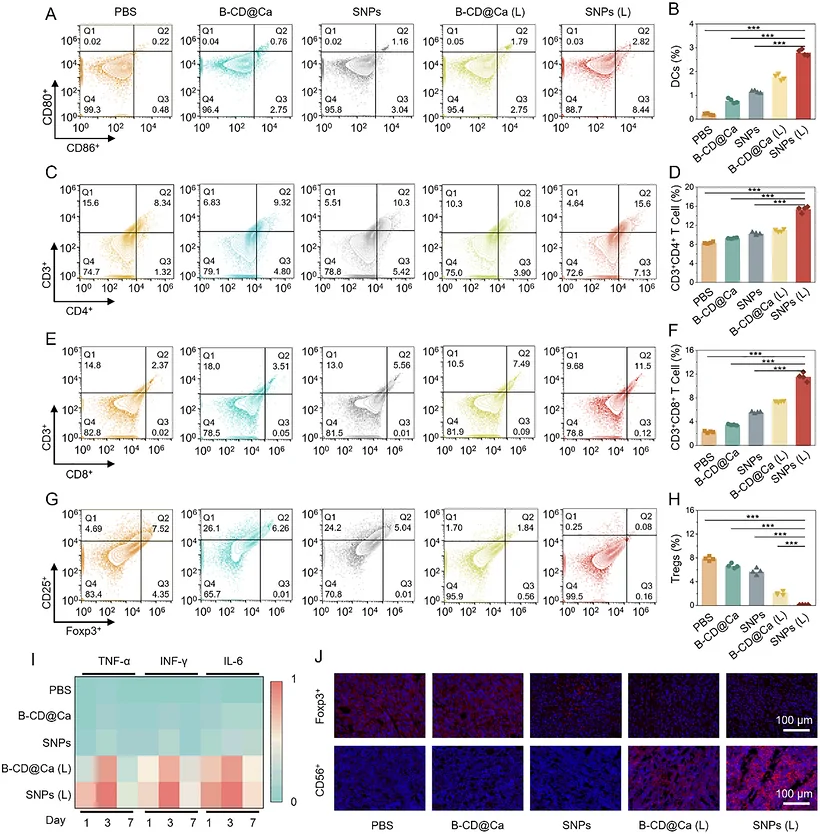
The SNPs‐induced immunogenic cell death orchestrates a comprehensive antitumor immune response, linking innate activation to adaptive immunity. (A,B) Flow cytometry analysis of DCs maturation in TDLNs. Flow cytometry analysis of (C,D) CD4^+^ T cells (CD3^+^CD4^+^), (E,F) CD8^+^ T cells (CD3^+^CD8^+^) and (G,H) Tregs (CD25^+^Foxp3^+^) in treated tumor tissues. (I) Serum levels of immunocompetent cytokines including IL‐6, IFN‐𝛾 and TNF‐𝛼 following different treatments. (J) Immunofluorescence staining of tumor‐infiltrating Foxp3^+^ Tregs and CD56^+^ NK cells across treatment groups. Data are presented as mean ± SD (n ≥ 3). Statistical significance was calculated via ordinary one‐way ANOVA with a Tukey's test. ^*^ *p* < 0.05; ^**^ *p* < 0.01; ^***^ *p* < 0.001; ^****^ *p* < 0.0001.

Regulatory T cells (Tregs) are immunosuppressive lymphocytes derived from CD4^+^ T helper cells that inhibit CTLs. Flow cytometry showed that the PBS group showed a high intratumoral Tregs proportion (CD25^+^Foxp3^+^, 7.5%), indicating an immunologically “cold” tumor environment. In contrast, SNPs (L) treatment remarkably reduced the Tregs proportion to 0.08% (Figure 5G,H), suggesting tumor microenvironment reprogramming via pyroptosis‐mediated immune activation. Consistent with flow cytometry results, immunofluorescent staining confirmed reduced Tregs infiltration in the SNPs (L) group (Figure 5J). Moreover, the CD8^+^/Tregs ratio was most significantly elevated in the SNPs (L) group (Figure S49), underscoring a shift toward an immunopermissive tumor milieu. Of note, SNPs (L) treatment also significantly enhanced intratumoral NK cell recruitment (Figure 5J), suggesting concurrent activation of innate immunity. Overall, this precisely targeted pyroptosis strategy successfully activated both adaptive and innate antitumor immune responses, reprogramming the ITM to achieve potent and systemic tumor regression.

### 2.7 Assessment of Tumor Metastasis Inhibition

Systemic antitumor immunity critically suppresses tumor recurrence and metastasis, ensuring favorable prognosis. We further evaluated SNPs (L) antitumor efficacy in a bilateral 4T1 tumor model (Figure 6A). As anticipated, SNPs (L) treatment efficiently suppressed primary tumor growth through precise pyroptosis induction (Figure 6B). The proportion of mature DCs was 9.73‐fold higher in the group of SNPs (L) compared to the PBS group (Figure 6D,E), implying robust DC activation for antitumor immunity. Notably, SNPs (L) treatment induced a potent abscopal effect, with 2 of 3 mice showing complete tumor regression of distant tumors by day 24 (Figure 6C and Figure S50), demonstrating significant activity against metastatic lesions. Immune profiles of these abscopal tumors exhibited markedly higher proportions of CD4^+^ (22.8%) and CD8^+^ T cells (10.3%) compared to other groups (Figure 6F,G and Figure S51), alongside significantly reduced Tregs accumulation (Figure 6H,I). These results convincingly evidenced that supramolecular pyroptosis activation remodeled the tumor immune microenvironment and induced systemic antitumor immunity, effectively controlling metastatic progression and improving overall therapeutic outcomes.

**FIGURE 6:**
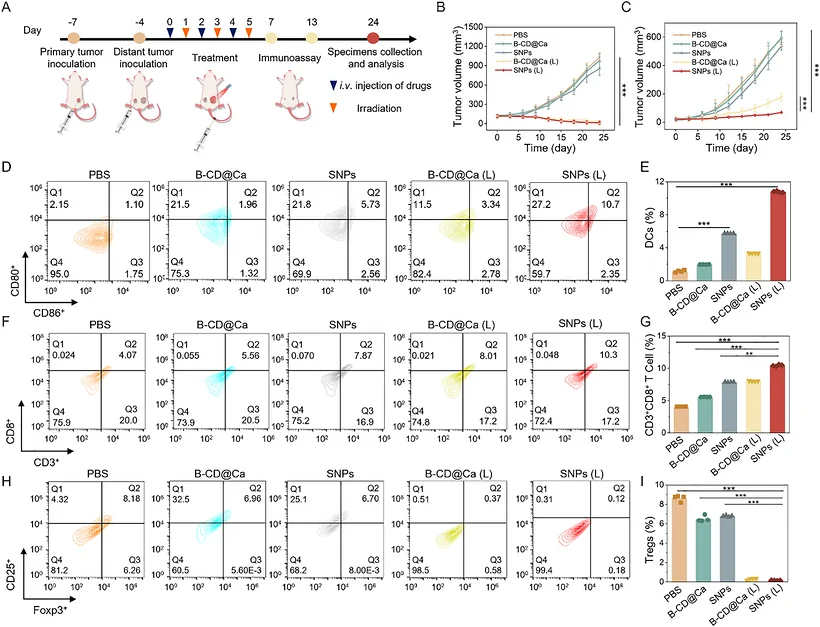
Anti‐metastasis efficacy and mechanism validation of the SNPs. (A) Therapeutic regimen for bilateral tumor therapy. Tumor growth curves of (B) primary and (C) abscopal tumors across different treatment groups. (D,E) Flow cytometry analysis of DCs maturation in TDLNs. Flow cytometry analysis of (F,G) CD8^+^ T cells (CD3^+^CD8^+^) and (H,I) Tregs (CD25^+^Foxp3^+^) in abscopal tumor tissues. Data are presented as mean ± SD (n ≥ 3). Statistical significance was calculated via ordinary one‐way ANOVA with a Tukey's test. ^*^ *p* < 0.05; ^**^ *p* < 0.01; ^***^ *p* < 0.001; ^****^ *p* < 0.0001.

## 3 Discussions

Pyroptosis, as a lytic and inflammatory form of programmed cell death, has recently emerged as a promising strategy for cancer immunotherapy. Unlike apotosis, pyroptosis induces plasma membrane rupture and the release of pro‐inflammatory cellular contents, thereby remodeling the ITM and activating robust antitumor immunity. Recent studies have demonstrated that GSDM family proteins, particularly GSDME, can be cleaved by caspase‐3 to form plasma membrane pores, serving as an executable pathway for pyroptosis. However, the precise and efficient induction of pyroptosis in tumor cells remains challenging, limiting its translational potential. Strategies that trigger pyroptosis with high specificity while minimizing the off‐target effects are urgently needed.

To address this, we developed a supramolecular nanoplatform that enables spatiotemporally controlled co‐delivery of Ca^2+^ and I_3_^−^, effectively addressing the persistent challenge of inducing potent and specific pyroptosis for cancer immunotherapy. While both Ca^2+^ overload and iodine‐base agents have shown therapeutic potential, their clinical translation has been hampered by inherent limitations, including tumor cell adaptive resistance, suboptimal pharmacokinetics, and systemic toxicity. Our design overcomes these challenges through a “disguise‐and‐release” strategy rooted in supramolecular engineering. Specifically, the host−guest encapsulation of I_3_^−^ within the hydrophobic cavity of *β*‐CD significantly enhanced its loading efficiency and physiological stability while masking systemic toxicity during circulation. In parallel, Ca^2+^ ions were electrostatically coordinated by carboxyl groups functionalized on the *β*‐CD surface and subsequently stabilized via in situ CaCO_3_ biomineralization, forming an acid‐responsive mineral shell. This dual‐confinement architecture ensures targeted co‐delivery of both agents while minimizing premature leakage and off‐target effects.

Upon cellular internalization, the developed SNPs exhibited a sophisticated, multi‐stage activation mechanism. Lysosomal acidity triggered the initial disassembly, dissolving the CaCO_3_ core to co‐release Ca^2+^ and I_3_^−^, a crucial priming step for subsequent therapeutic action. NIR irradiation then served as a precise secondary switch, driving the localized conversion of I_3_^−^ into highly cytotoxic I_2_. The resulting I_2_‐induced oxidative stress significantly impaired mitochondrial integrity, leading to the collapse of its calcium‐buffering capacity. This mitochondrial dysfunction, synergizing with exogenous Ca^2+^ release, overwhelmed cellular homeostasis and induced irreversible Ca^2+^ overload. Our experimental data provided direct evidence that this specific Ca^2+^ overload acted as the key trigger for caspase‐3/GSDME‐mediated pyroptosis. The ensuing pyroptotic cell lysis released substantial amounts of DAMPs, promoting dendritic cell maturation and cytotoxic T cell infiltration, thereby effectively converting the immunologically “cold” tumor microenvironment into a “hot” state conducive to antitumor immunity.

## 4 Conclusion

In summary, the constructed supramolecular thermal switch intelligently orchestrated a “mitochondrial dysfunction‐Ca^2+^ overload‐pyroptosis” cycle for cascade‐amplified cancer immunotherapy. The core innovation lied in the synergistic co‐delivery strategy that leveraged host−guest complexation and electrostatic interactions to achieve efficient loading and tumor‐specific release of both Ca^2+^ and the prodrug I_3_^−^. The NIR‐controlled conversion of I_3_^−^ to I_2_ initiates a well‐defined cascade involving oxidative stress and mitochondrial damage, ultimately disrupting endogenous calcium regulation and synergizing with exogenous Ca^2+^ to activate the caspase‐3/GSDME pyroptotic pathway. These findings not only validated Ca^2+^ overload as a specific and potent inducer of pyroptosis but also demonstrated that the resulting immunogenic cell death stimulated a robust antitumor immune response, effectively suppressing primary tumor growth and metastasis. Beyond its immediate therapeutic utility, the implemented supramolecular engineering approach establishes a versatile and generalizable platform. The modular design featuring a host for guest encapsulation, functional groups for ion coordination, and a biomineralization template, can be adapted for co‐delivery of other therapeutic pairs. Thus, this study opens a new avenue for precision oncology and provides a readily translatable strategy for advancing cancer immunotherapy.

## Author Contributions

**Dan Wu**, **Xinyang Yu** and **Shaolong Qi** conceived the project. **Jie Zhou**, **Borui Zhao** and **Yanrong Yang** performed the experiments. **Yibin Cao** and **Kunmin Ping** analyzed the results. **Chunyang Yu** performed the chemical computations. Dan Wu, Xinyang Yu, and Shaolong Qi wrote the manuscript.

## Ethics Statement

All animals were supplied by the Hangzhou Keling Biotechnology Co., Ltd. The execution of all animal experiments meticulously adhered to the Guidelines for the Care and Use of Laboratory Animals. All animal procedures and experiments were in conformity under the guidelines provided by the Animal Experimental Center of Zhejiang University of Technology (protocol number 20230719101).

## Conflicts of Interest

The authors declare no conflicts of interest.

## Supporting information




**Supporting File**: advs76664‐sup‐0001‐SuppMat.docx.

## Data Availability

All data associated with this study are present in the main text or Supporting Information. Additional data are available upon request to the authors.
