# Supplementary material for: A Supramolecular Thermal Switch for Precision Pyroptosis via Host−Guest Recognition and Electrostatic Interactions
Source: Adv Sci (Weinh). 2026 Jul 20:e76664. Online ahead of print. doi: 10.1002/advs.76664 (PMC13384033; doi:10.1002/advs.76664)
Supplement: Supplementary file 1 — Supporting File: advs76664‐sup‐0001‐SuppMat.docx. [file ADVS-9999-e76664-s001.docx]

Supporting Information

A Supramolecular Thermal Switch for Precision Pyroptosis *via* Host−Guest Recognition and Electrostatic Interactions

Dan Wu^*,†^ Jie Zhou,^†^ Yibin Cao,^†^ Kunmin Ping,^†^ Borui Zhao,^†^ Yanrong Yang,^†^ Chunyang Yu,^*,§^ Xinyang Yu,^*,‡^ and Shaolong Qi^*,‡^

^†^ State Key Laboratory of Advanced Separation Membrane Materials, Zhejiang Key Laboratory of Advanced Polymer Materials Modification and Application Technology, College of Materials Science and Engineering, Zhejiang University of Technology, Hangzhou 310014, P. R. China.

E-mail: [danwu@zjut.edu.cn](mailto:danwu@zjut.edu.cn)

^‡^ Ministry of Education Key Laboratory of Bioorganic Phosphorus Chemistry & Chemical Biology, Department of Chemistry, Tsinghua University, Beijing 100084, P. R. China.

E-mail: [yuxinyang@mail.tsinghua.edu.cn](mailto:guocanyu@mail.tsinghua.edu.cn); qishaolong@jlu.edu.cn

^§^ State Key Laboratory of Synergistic Chem-Bio Synthesis, School of Chemistry and Chemical Engineering, Shanghai Key Laboratory of Electrical Insulation and Thermal Aging, Shanghai Jiao Tong University, 800 Dongchuan Road, Shanghai, 200240, China.

E-mail: [chunyangyu@sjtu.edu.cn](mailto:chunyangyu@sjtu.edu.cn)

^*^ Corresponding authors.

1. **Materials and method**

**Materials**

All reagents were commercially available and used as supplied without further purification. Solvents were either employed as purchased or dried according to procedures described in the literature. CD-NH_2_ and BODIPY were synthesized according to literature procedures.^1,2^ ^1^H NMR spectra were recorded on a Bruker Avance DMX 400 spectrometer. 2D NOESY spectra were acquired on a Bruker Avance III-600 spectrometer. Fluorescence spectra were measured using an F-4600 FL spectrophotometer (Hitachi, Japan). UV-vis spectra were obtained with a UV-1900 spectrophotometer (Shimadzu, Japan). FTIR spectra were recorded on a Nicolet 6700 spectrometer (Thermo Fisher Scientific, USA). Isothermal titration calorimetry (ITC) was performed on a VP-ITC system (Malvern Panalytical, UK). Hydrodynamic size and zeta potential were determined using a Nano Brook Omni analyzer (Brookhaven Instruments, USA). X-ray diffraction (XRD) was performed on an Ultima IV diffractometer (Rigaku, Japan). X-ray photoelectron spectroscopy (XPS) was conducted on an ESCALAB220i-XL spectrometer (Thermo Fisher Scientific, UK). Transmission electron microscopy (TEM) images were acquired with an HT7800 microscope (Hitachi, Japan) operated at 120 kV. Elemental mapping images were acquired using a Talos F200X G2 field-emission transmission electron microscope (Thermo Scientific, Czech Republic) operating at 200 kV. The absorbance measurements in MTT, LDH, IL-1β, and IL-18 assays and chemiluminescence intensity measurements in ATP assay were performed using a Synergy H1 (Bio Tek, USA). HPLC analyses were carried out on an Agilent 1260 Infinity II system (Agilent Technologies Inc., USA). ICP-MS measurements were obtained with an ICP-9800 spectrometer (Shimadzu, Japan). Photothermal conversion experiments employed an FC-685LCPD-FC-2W laser (Ningbo Fingco Optoelectronics Tech Co., Ltd, China). Flow cytometry was performed on a BD FACS Canto II cytometer (Becton Dickinson, USA), and data were analyzed with FlowJo software. CLSM images were captured using an STELLARIS 5 (Leica, Germany). Bio-TEM observations were conducted with an H-7650 microscope (Hitachi, Japan). Fluorescence microscopy utilized a Nikon TS2-FL inverted microscope (Japan). *In vivo* fluorescence imaging was performed on an IVIS Spectrum system (PerkinElmer, USA). Western blot was carried on a Tanon 5200 imaging system (Tanon, China).

**Methods**

**Preparation of SNPs**

I_2_ (25 mg) dissolved in ethanol (0.3 mL) and KI (250 mg) dissolved in water (0.4 mL) were mixed and sonicated for 30 min to generate I_3_⁻ solution. Meanwhile, B-CD-COOH (350 mg) was dissolved in DMSO (25 mL) and sonicated for 1 h. Two solutions were mixed and stirred at 60 °C for 2 h. Then, CaCl_2_ solution (6.18 mg/mL, 20 mL) was added to the mixture and vigorously stirred for 12 h. Eventually, Na_2_CO_3_ solution (2.95, 5.9 or 11.8 mg/mL, 20 mL) was slowly added and further stirred for 12, 24 or 48 h. After vacuum freeze-drying, SNPs were obtained.

**Determination of the DLC and EE of I_3_^−^ in SNPs**

A standard curve of I_3_⁻ concentration versus absorbance was first established by UV-vis spectrophotometry (Figure S16). Then, 5.0 mL of the SNPs solution was dialyzed against 250 mL of PBS buffer using a 500 Da MWCO dialysis bag under continuous stirring for 12 hours. The I_3_⁻ concentration in the SNPs solution before and after dialysis were measured, allowing for the calculation of the DLC and EE using the formulas below:

DLC = $\frac{\text{Mass of encapsulated drug}}{\text{Mass of encapsulated drug}+\text{Mass of carrier}}$×100%

EE = $\frac{\text{Mass of encapsulated drug}}{\text{Mass of total drug}}$×100%

**Host**−**guest complexation between *β*-CD and I_3_^-^ measured by ^1^H NMR spectra**

Solutions of *β*-CD (1.0 mM) and I_3_⁻ (1.0 mM) + *β*-CD (1.0 mM) in DMSO-*d_6_* were prepared, respectively, and their ^1^H NMR spectra were measured on the Bruker AVANCE DMX 400 spectrometer.

**Morphological characterization of SNPs**

The hydrodynamic particle size and surface charge of SNPs were determined using dynamic light scattering, and the morphology of SNPs was examined using transmission electron microscope.

**Drug release profile of SNPs**

1.0 mL of SNPs aqueous solution (14.7 mM) was first irradiated at 1.0 W for 5 min and then transferred into a dialysis bag (500 Da MWCO). The sealed bag was immersed into a 50 mL centrifuge tube containing 39 mL of PBS buffer (*v*_DMF_/*v*_PBS_ = 3/7), and then oscillated at 37.0 °C under different conditions. 1.0 mL of dialysate was taken at the specified time point. At the same time, 1.0 mL of fresh PBS buffer (*v*_DMF_/*v*_PBS_ = 3/7) was added back into dialysate. The release profile of Ca^2+^ was monitored by ICP-MS, and the release profile of I_3_^-^ was supervised by UV-vis spectroscopy.

**Evaluation of the photothermal conversion efficiency of SNPs**

The photothermal performance of SNPs was evaluated under continuous irradiation with a 670 nm laser at a constant power density (1.0 W·cm^-2^). Temperature variations over time were recorded in real time using a Flir One Pro infrared thermal imaging camera. Four consecutive heating-cooling cycles were then performed to assess photothermal stability. The maximum temperature increment (ΔTₘₐₓ) and the dimensionless driving force temperature (θ) were determined based on the initial ambient temperature, the maximum equilibrium temperature under irradiation, and the corresponding time parameters. Linear fitting of –ln(θ) against cooling time yielded the heat transfer coefficient (*hS*) of the system. The absorbance of the samples at 670 nm was measured using a UV-vis spectrophotometer. Finally, the photothermal conversion efficiency (*η*) of SNPs was calculated using the following equation:

$$\eta=\frac{hS\cdot\Delta T_{\text{max}}}{P_{\text{laser}}\cdot(1-{10}^{-A})}$$

where *η* is the photothermal conversion efficiency (dimensionless, usually expressed as a percentage), $hS$ is the heat transfer coefficient (W/°C), $\Delta T_{\text{max}}$ is the maximum temperature increase under irradiation (°C), $P_{\text{laser}}$ is the laser power (W), and $A$ is the absorbance of the sample at the laser wavelength (dimensionless). The heat transfer coefficient $hS$ was further calculated as $hS=mC/\tau_{s}$, with $m$ being the solution mass (g), $C$ the specific heat capacity of the solution (J/g·°C), and $\tau_{s}$ the system time constant. Using these parameters, the photothermal conversion efficiency of SNPs was determined to be approximately 60.8%.

**Cell uptake of SNPs**

First, 4T1 cells were seeded in 24-well plates at a density of 5 × 10^4^ cells/well, and allowed to adhere overnight. Then, 4T1 cells were cultured with SNPs solutions (11.0 μM) for 1, 2, 4, 8 and 12 h, respectively. After treatment, the cells were then stained with Lyso-tracker for 45 min, washed with PBS, and fixed with 4.0% paraformaldehyde for 15 min. The fixed cells were then stained with DAPI for 5 min. Eventually, cell images were taken using a confocal laser scanning microscope.

**Evaluation of cytotoxicity**

First, 4T1 cells were seeded in 96-well plates at a density of 1 × 10^4^ cells/well, and allowed to adhere overnight. Then different formulations were cultured with 4T1 cells for 12 h, followed by 5 min irradiation of 670 nm laser (1.0 W) when necessary. After another 12 h incubation, the medium was removed and 100 μL of fresh MTT solution (0.5 mg/mL) was added to each well. After incubation for 4 h, MTT solution was removed and 150 μL of DMSO was added to each well. Eventually, the UV absorbance of the formazan product at 490 nm was measured using a microplate reader.

**Live/dead dual-staining**

First, 4T1 cells were seeded in 96-well plates at a density of 1 × 10^4^ cells/well, and allowed to adhere overnight. Then different formulations were cultured with 4T1 cells for 12 h, followed by 5 min irradiation of 670 nm laser (1.0 W) when necessary. After another 12 h incubation, cells were washed with PBS and stained with calcein acetoxymethyl ester (Calcein-AM) (2.0 μM) and propidium iodide (PI) (5.0 μM) in the dark for 20 min at 37 °C. The cell imaging was taken on an inverted fluorescent microscope.

**Programmed cell death analysis**

First, 4T1 cells were seeded in 6-well plates at a density of 2.5 × 10^5^ cells/well, and allowed to adhere overnight. Then different formulations were cultured with 4T1 cells for 12 h, followed by 5 min irradiation of 670 nm laser (1.0 W) when necessary. After another 12 h incubation, the cells were harvested, washed by PBS, resuspended in 1.0 mL of PBS, and stained with fluorescein isothiocyanate (Annexin V-FITC) (20 μL) and PI (50 μL) in the dark for 20 min. The programmed cell death manner of cells was analyzed using a flow cytometer (excitation wavelength: 490 nm for Annexin V-FITC and 535 nm for PI).

**Intracellular ROS detection**

4T1 cells were seeded in 96-well plates at a density of 1 × 10^4^ cells/well, and allowed to adhere overnight. Then different formulations were cultured with 4T1 cells for 12 h, followed by 5 min irradiation of 670 nm laser (1.0 W) when necessary. After another 12 h incubation, the culture media were freshly replaced and 2′,7′-dichlorofluorescin diacetate (DCFH-DA) was added to each well. After 30 min incubation at 37 ^o^C, intracellular ROS level was analyzed using confocal laser scanning microscope.

**JC-1 staining**

4T1 cells were seeded in 96-well plates at a density of 1 × 10^4^ cells/well, and allowed to adhere overnight. Then different formulations were cultured with 4T1 cells for 12 h, followed by 5 min irradiation of 670 nm laser (1.0 W) when necessary. After another 12 h incubation, the culture medium was freshly replaced, and JC-1 staining working solution (100 μL) was added to each well. After 30 min incubation at 37 ^o^C, the cells were washed with JC-1 staining buffer. Subsequently, 100 μL of cell culture medium was added and cell imaging was taken using a confocal laser scanning microscope.

**Bio-TEM imaging**

4T1 cells were seeded in 6-well plates at a density of 2.5 × 10^5^ cells/well, and allowed to adhere overnight. Then different formulations were cultured with 4T1 cells for 12 h, followed by 5 min irradiation of 670 nm laser (1.0 W) when necessary. After another 12 h incubation, cells were washed with PBS and collected for bio-TEM imaging.

**Intracellular Ca^2+^ detection**

4T1 cells were seeded in 96-well plates at a density of 1 × 10^4^ cells/well, and allowed to adhere overnight. Then different formulations were cultured with 4T1 cells for 12 h, followed by 5 min irradiation of 670 nm laser (1.0 W) when necessary. After another 12 h incubation, the culture media were freshly replaced and Fluo-4 AM was added to each well. After 30 min incubation at 37 ^o^C, intracellular Ca^2+^ level was analyzed using confocal laser scanning microscope.

**Intracellular LPO analysis**

4T1 cells were seeded in 96-well plates at a density of 1 × 10^4^ cells/well, and allowed to adhere overnight. Then different formulations were cultured with 4T1 cells for 12 h, followed by 5 min irradiation of 670 nm laser (1.0 W) when necessary. After another 12 h incubation, the culture media were freshly replaced and Liperfluo was added to each well. After 30 min incubation at 37 ^o^C, intracellular LPO level was analyzed using confocal laser scanning microscope.

**IL-1β and IL-18 secretion**

4T1 cells were seeded in 96-well plates at a density of 1 × 10^4^ cells/well, and allowed to adhere overnight. Then different formulations were cultured with 4T1 cells for 12 h, followed by 5 min irradiation of 670 nm laser (1.0 W) when necessary. After another 12 h incubation, the levels of IL-1β and IL-18 in media were analyzed using ELISA kit.

**LDH level analysis**

4T1 cells were seeded in 96-well plates at a density of 1 × 10^4^ cells/well, and allowed to adhere overnight. Then different formulations were cultured with 4T1 cells for 12 h, followed by 5 min irradiation of 670 nm laser (1.0 W) when necessary. After another 12 h incubation, the level of LDH in media was analyzed using LDH assay kit.

**Western blot**

4T1 cells were seeded in 6-well plates at a density of 2.5 × 10^5^ cells/well, and allowed to adhere overnight. Then different formulations were cultured with 4T1 cells for 12 h, followed by 5 min irradiation of 670 nm laser (1.0 W) when necessary. After another 12 h incubation, cells were lysed in RIPA buffer with a protease inhibitor cocktail. Then, proteins were quantified through Pierce BCA Protein Assay Kit and denatured at 95 °C for 5 min in Laemmli buffer with 150 mM DTT. After denaturation, an equal number of proteins (20 µg) was loaded in 4 to 15% SDS-PAGE and transferred to a 0.45 pore polyvinylidene difluoride filter. Polyclonal rabbit anti-GSDME-N (1:1000 dilution overnight at 4 °C), rabbit anti-caspase-3 (1:1000 dilution overnight at 4 °C), rabbit anti-PP2A (1:500 dilution overnight at 4 °C) and mouse monoclonal anti-β-actin (1:5000 dilution overnight at 4 °C) antibodies were used after saturation in 3% bovine serum albumin. After incubation with horseradish peroxidase (HRP)-linked secondary antibody (1:3000 dilution 1 h at room temperature), Western Lightning Chemiluminescence Reagent Plus was used for visualization. Images were acquired with the Tanon 5200 imaging system and analyzed with ImageLab software.

**HMGB1 staining**

4T1 cells were seeded in 96-well plates at a density of 1 × 10^4^ cells/well, and allowed to adhere overnight. Then different formulations were cultured with 4T1 cells for 12 h, followed by 5 min irradiation of 670 nm laser (1.0 W) when necessary. After another 12 h incubation, cells were fixed with 4% paraformaldehyde, and stained with HMGB1 primary antibody overnight, goat Alexa Fluor 488 anti-Mouse IgG secondary antibody for 1 h and DAPI staining for 5 min, respectively. The cell images were taken using a confocal laser scanning microscope.

**CRT staining**

4T1 cells were seeded in 96-well plates at a density of 1 × 10^4^ cells/well, and allowed to adhere overnight. Then different formulations were cultured with 4T1 cells for 12 h, followed by 5 min irradiation of 670 nm laser (1.0 W) when necessary. After another 12 h incubation, cells were fixed with 4% paraformaldehyde, and stained with CRT primary antibody overnight, goat Alexa Fluor 488 anti-Mouse IgG secondary antibody for 1 h and DAPI staining for 5 min, respectively. The cell images were taken using a confocal laser scanning microscope.

**ATP level analysis**

4T1 cells were seeded in 96-well plates at a density of 1 × 10^4^ cells/well, and allowed to adhere overnight. Then different formulations were cultured with 4T1 cells for 12 h, followed by 5 min irradiation of 670 nm laser (1.0 W) when necessary. After another 12 h incubation, the level of ATP in media was analyzed using ATP assay kit.

**Pyroptosis inhibitor control experiment**

4T1 cells were seeded in confocal culture dishes at 1 × 10^5^ cells per well and allowed to adhere overnight. Cells were then pre-treated with the pyroptosis inhibitor Z-DEVD-FMK for 2 h, followed by incubation with SNPs solution for 12 h. Where indicated, cells were irradiated with a 670 nm laser (1.0 W) for 5 min. At 2 h post-irradiation, pyroptotic morphological changes were observed and recorded under the bright-field channel of a confocal microscope. After an additional 10 h incubation, cells were fixed with 4% paraformaldehyde, permeabilized, and blocked. Cells were then incubated overnight at 4 °C with primary antibodies against CRT or HMGB1, followed by incubation with corresponding Alexa Fluor 488-conjugated secondary antibodies for 1 h at room temperature. Nuclei were counterstained with DAPI for 5 min, and fluorescence images were acquired using a confocal microscope.

**Pharmacokinetic study**

Healthy SD rats (200 g) were randomly divided into 4 groups and intravenously injected with 1.0 mL of PTX (1.0 mg/kg), TPPS (1.0 mg/kg), B-CD-COOH (2.0 mg/kg) or SNPs (12.0 mg/kg), respectively. At the determined time points, blood samples (300 μL) were collected from orbit and centrifuged for 5 min (4000 rpm). The resulting serums were then mixed with 300 μL of acetonitrile and centrifuged at 4000 rpm for 5 minutes to obtain the supernatant. Accordingly, serum concentrations of PTX and TPPS were quantified by HPLC, while the BODIPY content in B-CD-COOH and SNPs groups was measured by UV-vis spectroscopy. A two-compartment pharmacokinetic model was used to calculate the pharmacokinetic parameters. The drug-time curve equations for the four groups were as follows:

PTX: $C(t)=96.1e^{-2.41t}+3.9e^{-0.148t}$

TPPS: $C(t)=94.2e^{-1.54t}+5.8e^{-0.0211t}$

B-CD-COOH: $C(t)=53.8e^{-0.789t}+10.27e^{-0.0779t}$

SNPs: $C(t)=60.3e^{-0.72t}+20.5e^{-0.05t}$

From these equations, the central half-life ($t_{1/2\alpha}$), peripheral elimination half-life ($t_{1/2\beta}$), and area under the concentration-time curve (AUC) were determined.

***In vivo* fluorescence imaging**

4T1 tumor-bearing mice were intravenously administered with 200 μL of SNPs solution (6.0 mg kg^- 1^). At the determined time points, mice were anesthetized and imaged by an *in vivo* imaging system (λ_ex_ = 710 nm, λ_em_ = 760 nm). After *in vivo* imaging, mice were euthanized, and main organs (thyroid, heart, liver, spleen, lung, kidney) and tumor tissues were collected for *ex vivo* imaging.

**Tissue distribution of I_2_**

1 × 10^6^ 4T1 cells suspended in 100 μL of PBS were subcutaneously implanted into the left flank abdomen of BALB/c mice (7 weeks). When tumor volumes reached 100 mm^3^, mice were randomly divided into 3 groups (*n* = 4) and were intravenously administered with 100 μL of PBS, I_2_ (2.0 mg kg^-1^), or SNPs (12.0 mg kg^-1^) every 3 days for a total of three doses. For the illumination group, mice were irradiated with 670 nm laser (1.0 W) for 5 min at 8 h post-injection. After treatments, the mice were euthanized, and main organs (heart, liver, spleen, lung, kidney) and tumor tissues were collected. The tissues were homogenized in PBS and filtered to obtain single-cell suspensions. The I_2_ content in these suspensions was then determined by UV-vis spectrophotometry.

**Antitumor therapy**

1 × 10^6^ 4T1 cells suspended in 100 μL of PBS were subcutaneously implanted into the left flank abdomen of BALB/c mice (7 weeks). When tumor volumes reached 100 mm^3^, mice were randomly divided into 5 groups (*n* = 4) and were intravenously administered with 100 μL of PBS, B-CD@Ca (10.0 mg kg^-1^), SNPs (12.0 mg kg^-1^), B-CD@Ca (L) (10.0 mg kg^-1^) or SNPs (L) (12.0 mg kg^-1^) every 3 days for a total of three doses. For the illumination group, mice were irradiated with 670 nm laser (1.0 W) for 5 min at 8 h post-injection. The tumor size was measured every 3 days and tumor volume *V* was calculated according to the formula *V* = (tumor length) × (tumor width)2/2. After treatments, mice were euthanized, and main organs (heart, liver, spleen, lung, kidney, tumor-draining lymph node) and tumor tissues were collected. Tumors were homogenized in PBS and filtered to obtain single-cell suspension which were stained by anti-CD3ε-PE, anti-CD4-FITC, anti-CD8a-PE-Cy7, anti-CD25-APC or anti-FoxP3-PE and analyzed by flow cytometry. Meanwhile, the excisional tumors were immunofluorescently stained with H&E, Ki67, CRT, HMGB1, Foxp3 and CD56, and further analyzed with WB to study the pyroptotic protein expressions. Besides, tumor-draining lymph nodes were homogenized in PBS and filtered to obtain single-cell suspension which were stained with anti-CD11c-FITC, anti CD80-PE and anti-CD86-APC antibodies and analyzed by flow cytometry. During treatment, blood samples of mice were collected on day 1, 3 and 7, and the levels of TNF-α, INF-γ and IL-6 were analyzed by ELISA Kit.

**Acute toxicity: hematological analysis, liver and kidney function, and histopathological examination**

Nine healthy 7-week-old BALB/c mice were randomly divided into three groups (*n* = 3) and received a single intravenous injection of PBS, SNPs at 12 mg/kg, or SNPs at 60 mg/kg (100 μL). At predetermined time points, blood samples were collected *via* retro-orbital puncture. Each sample was divided into two aliquots: one was analyzed using an automated hematology analyzer for complete blood count, and the other was centrifuged at 4000 rpm for 5 minutes to obtain plasma, which was then analyzed using an automated biochemistry analyzer to assess liver and kidney function. Body weights were recorded on days 0, 3, 6, 9, 12, and 15. On day 15, mice were euthanized, and the heart, liver, spleen, lung, and kidney were harvested for histopathological examination using H&E staining.

**Long-term toxicity: hematological analysis, histopathological examination, and histamine assay**

Eight healthy 7-week-old BALB/c mice were randomly divided into two groups (*n* = 4) and received a total of three intravenous injections (every 3 days) of either PBS or SNPs at 12 mg/kg (100 μL). After the final injection, body temperature was measured at 0.5 h and 6 h. Blood samples collected *via* retro-orbital puncture at predetermined time points were divided into two aliquots: one was analyzed for complete blood count using an automated hematology analyzer, and the other was used to determine histamine concentration with a commercial assay kit. On day 30, mice were euthanized, and the heart, liver, spleen, lung, and kidney were harvested for histopathological evaluation using H&E staining.

**Thyroid tissue sectioning and H&E staining**

Six healthy 7-week-old BALB/c mice were randomly divided into two groups (*n* = 3) and received three intravenous injections (every 3 days) of either PBS or SNPs at 12 mg/kg (100 μL). After the final administration, mice were euthanized, and thyroid tissues were harvested for histopathological analysis using H&E staining.

**Immunogenic response *in vivo***

1 × 10^6^ 4T1 cells suspended in 100 μL of PBS were subcutaneously implanted into the left flank abdomen of BALB/c mice (7 weeks). When tumor volumes reached 100 mm^3^, mice were randomly divided into 3 groups (*n* = 4) and were intravenously administered with 100 μL of PTX (1.0 mg/kg), TPPS (1.0 mg/kg), or SNPs (12.0 mg kg^-1^) every 3 days for a total of three doses. For illumination group, mice were irradiated with 670 nm laser (1.0 W) for 5 min at 8 h post-injection. During treatment, blood samples of mice were collected on day 1, 3 and 7, and the levels of TNF-α, INF-γ and IL-6 were analyzed by ELISA Kit.

**Anti-metastasis therapy**

Three days after injecting 1 × 10^6^ 4T1 cells suspended in PBS into the left flank abdomen of BALB/c mice, 1 × 10^6^ 4T1 cells were injected subcutaneously into the right flank of mice to plant the distant tumors. Four days later, tumor-bearing mice were divided randomly into 5 groups (*n* = 3) and were intravenously administered with 100 μL of PBS, B-CD@Ca (10.0 mg kg^-1^), SNPs (12.0 mg kg^-1^), B-CD@Ca (L) (10.0 mg kg^-1^) or SNPs (L) (12.0 mg kg^-1^) every 3 days for a total of three doses. For illumination group, mice were irradiated with 670 nm laser (1.0 W) for 5 min at 8 h post-injection. The tumor size was measured every 3 days and tumor volume *V* was calculated according to the formula *V* = (tumor length) × (tumor width)2/2. After treatments, mice were euthanized, and tumor-draining lymph node and tumor tissues were collected. Tumors were homogenized in PBS and filtered to obtain single-cell suspension which were stained by anti-CD3ε-PE, anti-CD4-FITC, anti-CD8a-PE-Cy7, anti-CD25-APC or anti-FoxP3-PE and analyzed by flow cytometry. Meanwhile, tumor-draining lymph nodes were homogenized in PBS and filtered to obtain single-cell suspension which were stained with anti-CD11c-FITC, anti CD80-PE and anti-CD86-APC antibodies and analyzed by flow cytometry.

**Statistics**

Data are presented as mean ± SD (n ≥ 3). Statistical significance was calculated *via* ordinary one-way ANOVA with a Tukey’s test. **p* < 0.05; ***p* < 0.01; ****p* < 0.001; *****p* < 0.0001.

**
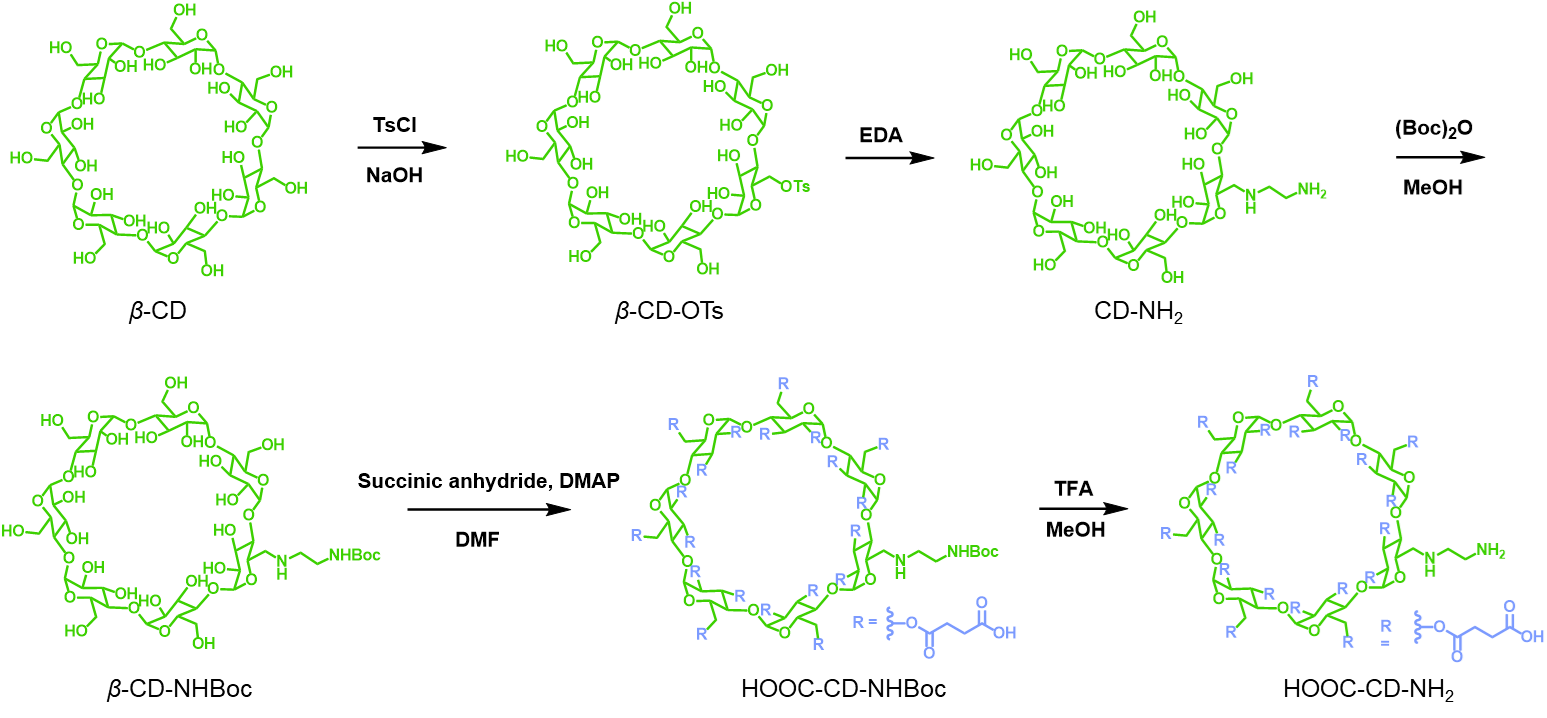
**

**Figure S1.** Synthetic route of CD-NH_2_.

**Synthesis of *β*-CD-OTs**

*β*-CD (20.0 g, 17.6 mmol) was first suspended in 167 mL of water, then NaOH (2.19 g, 54.7 mmol) dissolved in 7.0 mL of water was added dropwise. The mixture was immersed into the ice-water bath, and TsCl (5.04 g, 26.4 mmol) dissolved in 10 mL of acetonitrile was dripped slowly. After 2 h, the mixture was refrigerated overnight at 4 ^o^C. The resulting precipitate was collected *via* suction filtration and recrystallized in hot water for three times. ^1^H NMR (400 MHz, DMSO-*d*_6_) δ 7.77–7.75 (d, 2H), 7.45–7.43 (d, 2H), 5.74 (m, 14H), 4.84–4.78 (m, 6H), 4.52 (m, 6H), 4.20 (m, 1H), 3.66–3.20 (m, 42H), 2.44 (s, 3H).

**Synthesis of CD-NH_2_**

*β*-CD-OTs (5.0 g, 3.88 mmol) was dissolved in 30 mL ethylenediamine, and stirred at 70 ^o^C. After 24 h, the mixture was cooled down and poured into acetone (100 mL). The precipitate was collected *via* centrifugation and washed with acetone for three times. ^1^H NMR (400 MHz, DMSO-*d*_6_) δ 5.73 (m, 14H), 4.91–4.76 (m, 7H), 4.75–4.17 (m, 6H), 3.79–3.45 (m, 28H), 3.33–3.28 (m, 12H), 3.17 (s, 1H), 3.00–2.63 (m, 5H).

**Synthesis of CD-NHBoc**

CD-NH_2_ (3.7 g, 3.0 mmol) was first dissolved in methanol (100 mL) containing triethylamine (1.01 g, 10.0 mmol), then di-tert-butyl dicarbonate (4.4 g, 20.0 mmol) was added dropwise under the ice-water bath. The mixture was stirred at room temperature for 24 h. After reaction finished, the solvent was removed using a rotary evaporator and the crude product was poured into acetone (100 mL). The precipitate was collected *via* centrifugation, washed with acetone (3 × 100 mL) and dried under vacuum to afford CD-NHBoc with a yield of 85.7%. ^1^H NMR (400 MHz, DMSO-*d*_6_) δ 6.76 (m, 1H), 5.97-5.68 (m, 14H), 4.90-4.84 (m, 7H), 4.56-4.46 (m, 5H), 3.55 (m, 29H), 3.30-3.28 (m, 14H), 3.06-2.83 (m, 5H), 1.46-1.31 (m, 9H).

**Synthesis of HOOC-CD-NHBoc**

CD-NHBoc (2.7 g, 2.0 mmol), succinic anhydride (6.0 g, 60.0 mmol) and 4-dimethylaminopyridine (122 mg, 1.0 mmol) were dissolved in DMF (50 mL) and stirred at 65 °C for 12 h. After cooling to room temperature, the crude product was poured into diethyl ether (400 mL). The precipitate was collected *via* centrifugation, washed with acetone (3 × 100 mL) and dried under vacuum to afford HOOC-CD-NHBoc with a yield of 92.1% yield. ^1^H NMR (400 MHz, DMSO-*d*_6_) δ 5.44-5.01 (m, 7H), 4.93-4.59 (m, 7H), 4.40-3.47 (m, 42H), 2.64-2.46 (m, 60H), 1.54-1.36 (m, 8H).

**Synthesis of HOOC-CD-NH_2_**

HOOC-CD-NHBoc (3.1 g, 2.0 mmol) was dissolved in methanol (50 mL), and then was added with 3 mL of trifluoroacetic acid. The mixture was stirred at room temperature for 4 h. After removing solvent using a rotary evaporator, the crude product was poured into acetone (100 mL). The precipitate was collected *via* centrifugation, washed with diethyl ether (3 × 100 mL) and dried under vacuum to afford HOOC-CD-NH_2_ with a yield of 96.4% yield. ^1^H NMR (400 MHz, DMSO-*d*_6_) δ 5.47-5.06 (m, 7H), 4.88-4.64 (m, 7H), 3.42-3.18 (m, 42H), 2.74-2.41 (m, 60H).

**Synthesis of B-CD-COOH**

BODIPY (3.9 g, 6.0 mmol) and 1,1'-carbonyldiimidazole (1.17 g, 7.2 mmol) were dissolved in dichloromethane (50 mL) and stirred at room temperature for 4 h. After removing solvent using a rotary evaporator, the product was added with HOOC-CD-NH_2_ (3.0 g, 2.00 mmol) dissolved in DMSO (50 mL) and 20 μL of triethylamine. The mixture was stirred at room temperature for 24 h. After reaction finished, the mixture was washed with dichloromethane (3 × 100 mL) and diethyl ether (3 × 100 mL), and dried under vacuum to afford B-CD-COOH with a yield of 53.4%. ^1^H NMR (400 MHz, DMSO-*d*_6_) δ 7.46-7.41 (m, 2H), 7.30 (m, 2H), 7.08-6.87 (m, 2H), 6.81-6.79 (m, 1H), 5.26-4.96 (m, 7H), 4.81-4.61 (m, 7H), 3.89-3.22 (m, 42H), 3.00 (m, 5H), 2.60-2.50 (m, 60H), 1.59-1.36 (m, 3H).

**Figure S2.** ^1^H NMR spectrum of *β*-CD-OTs.

**Figure S3.** ^1^H NMR spectrum of CD-NH_2_.


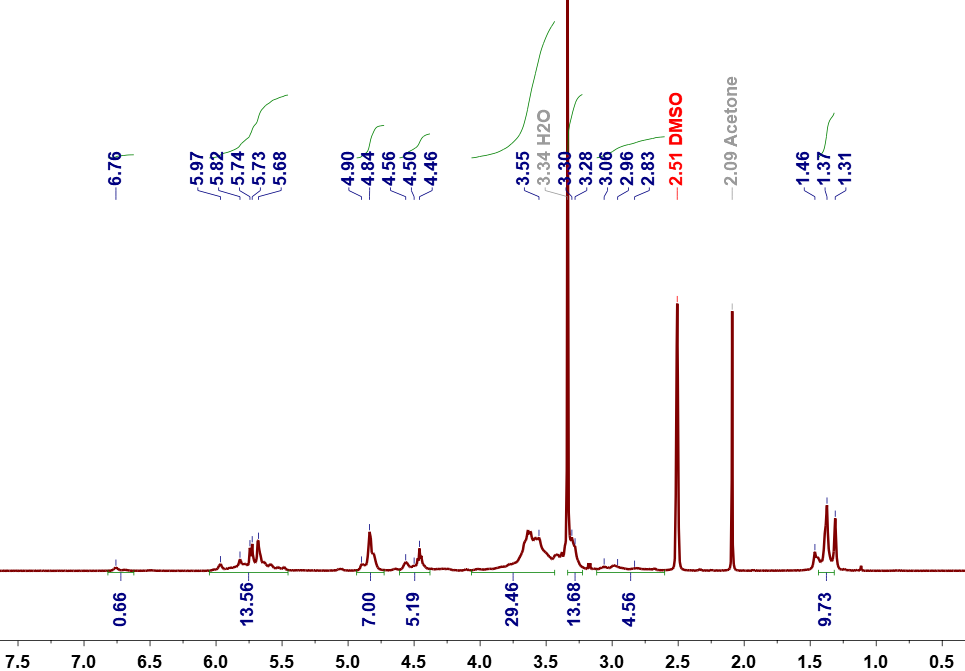


**Figure S4.** ^1^H NMR spectrum of CD-NHBoc.


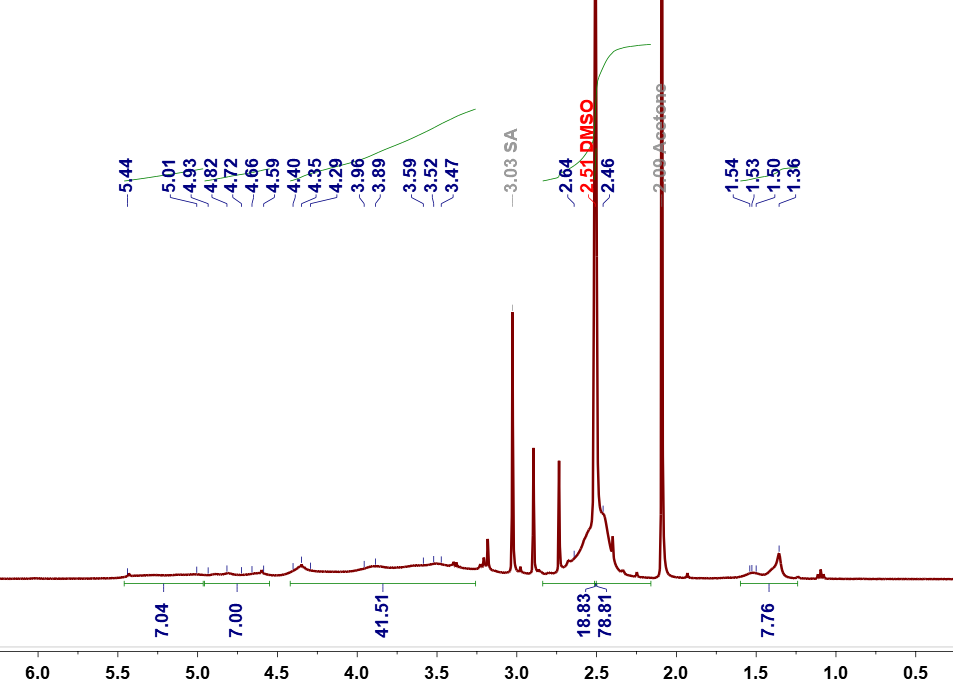


**Figure S5.** ^1^H NMR spectrum of HOOC-CD-NHBoc.


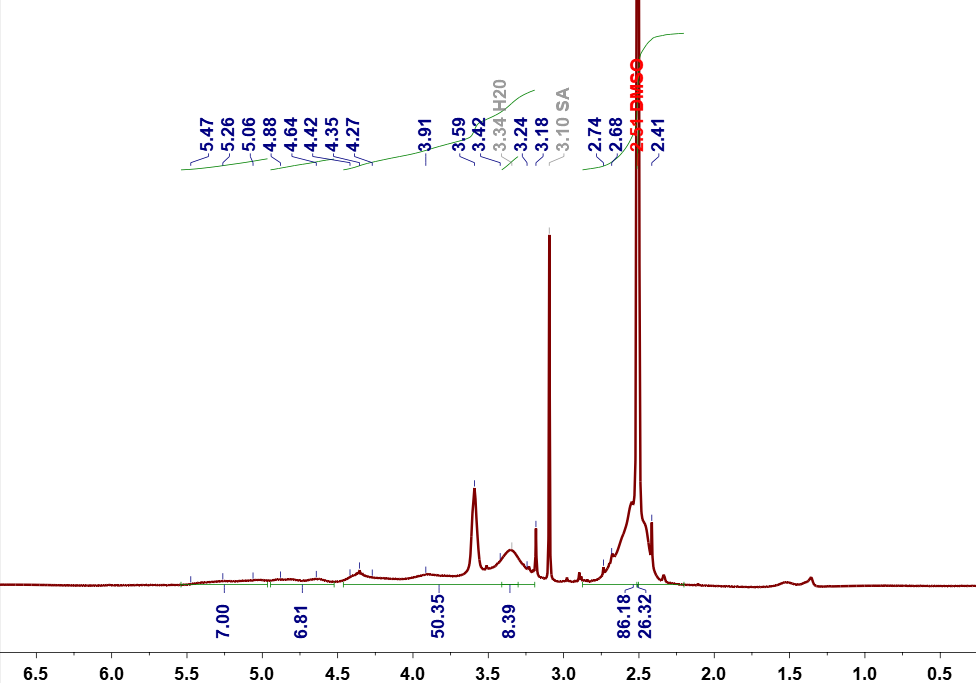


**Figure S6.** ^1^H NMR spectrum of HOOC-CD-NH_2_.

**Figure S7.** MS spectrum of HOOC-CD-NH_2_.


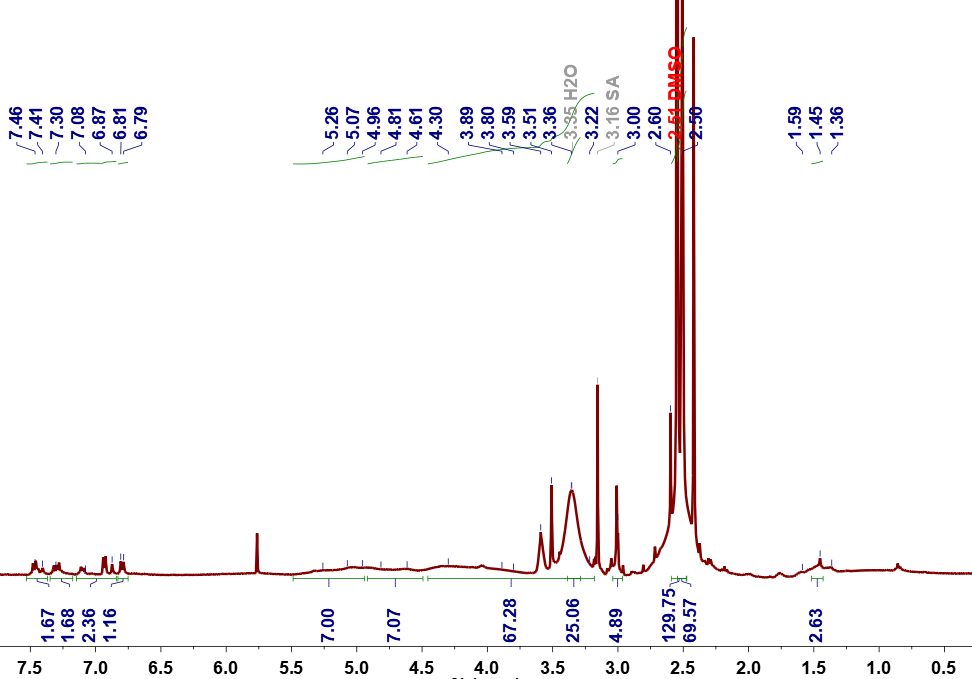


**Figure S8.** ^1^H NMR spectrum of B-CD-COOH.

**Figure S9.** Synthetic route of BODIPY.^2^


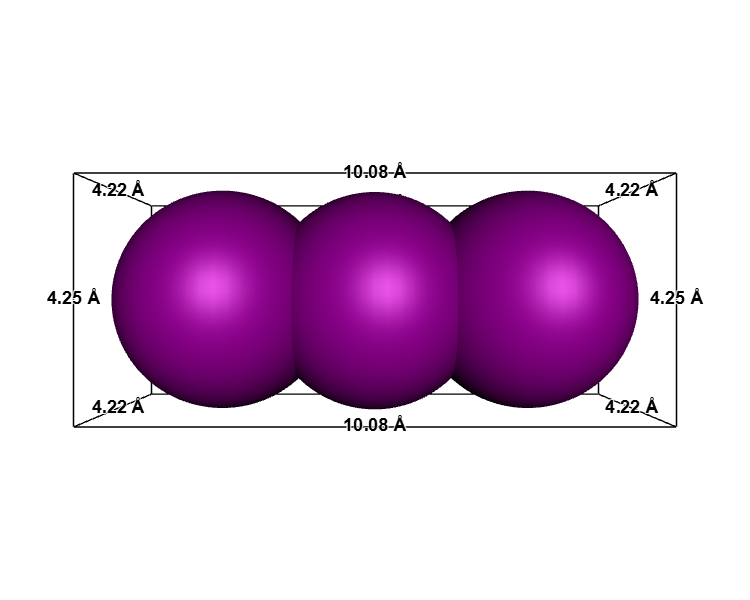


**Figure S10.** Calculated molecular dimension of I_3_^−^ based on Chemdraw soft.

| ITC Data Parameters | |
| --- | --- |
| *N* | 1.00 ± 0 Sites |
| *K*_a_ | 1.62E3 ± 18.9 M^-1^ |
| $\Delta$*H* | -8597 ± 42.79 cal/mol |
| $\Delta$*S* | -14.1 cal/mol/deg |

**Figure S11.** Thermodynamic information of host-guest complexation revealed by ITC experiment.





**Figure S12.** PDI values of nanoparticles under varying biomineralization conditions.


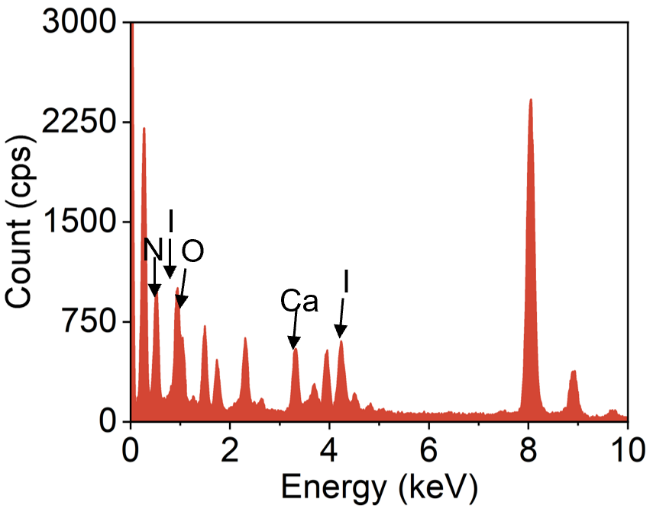


**Figure S13.** EDX spectroscopy of SNPs.


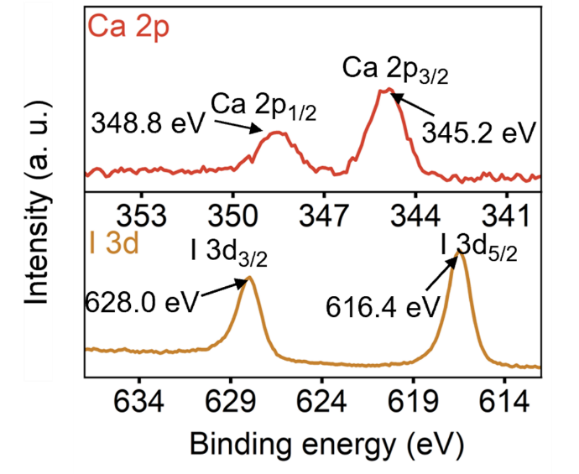


**Figure S14.** XPS spectra of SNPs.





**Figure S15.** The average diameter of SNPs after incubation in H_2_O, PBS, RPMI 1640 and 10% FBS for 24 h.


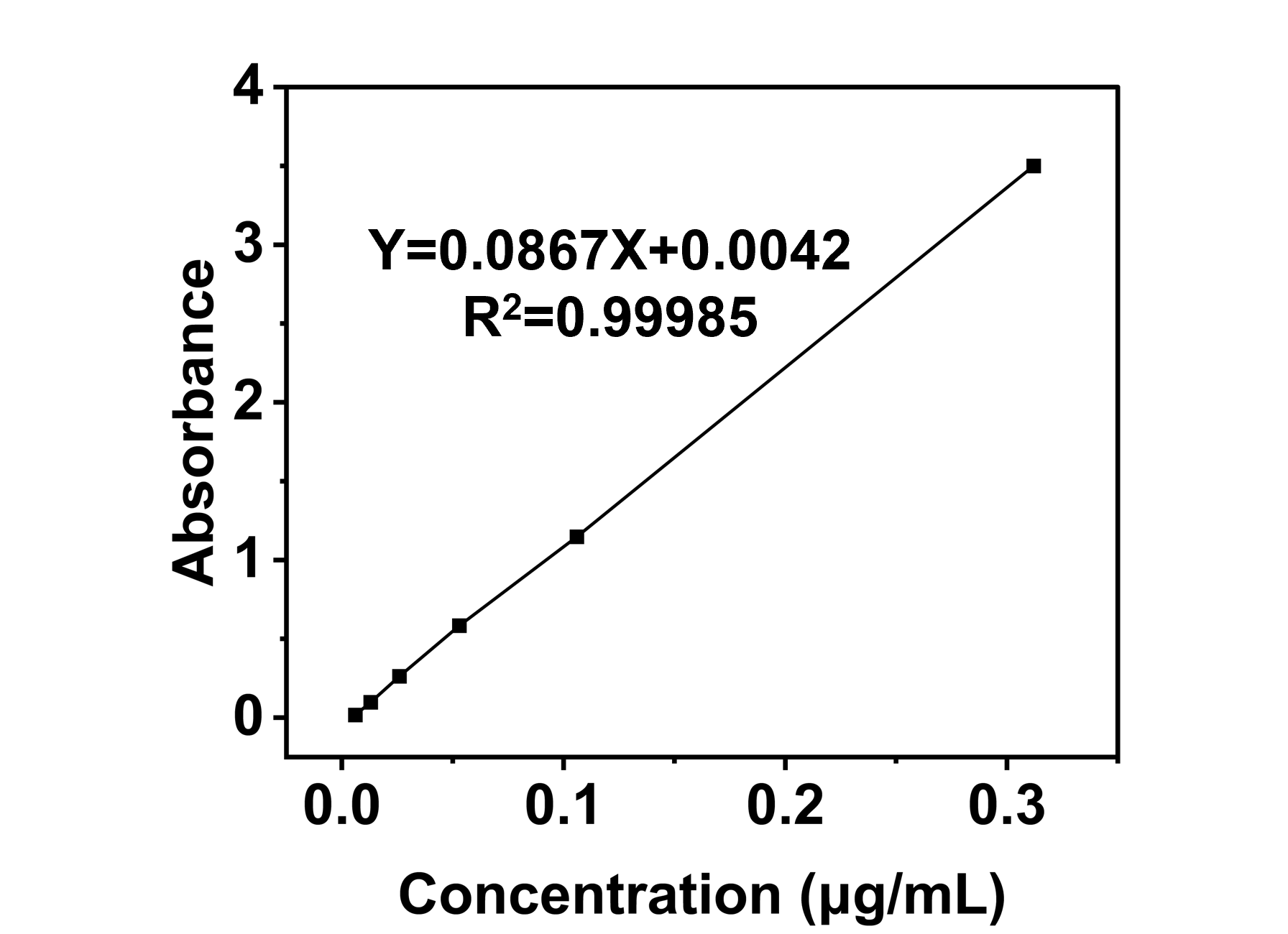


**Figure S16.** Standard curve of I_3_^−^ concentration versus absorbance.


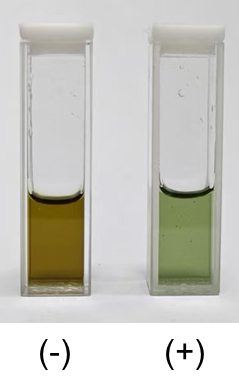


**Figure S17.** The color change of SNPs aqueous solution before (–) and after (+) NIR irradiation.

**Figure S18.** Temperature profiles of SNPs during multiple on/off laser cycles.


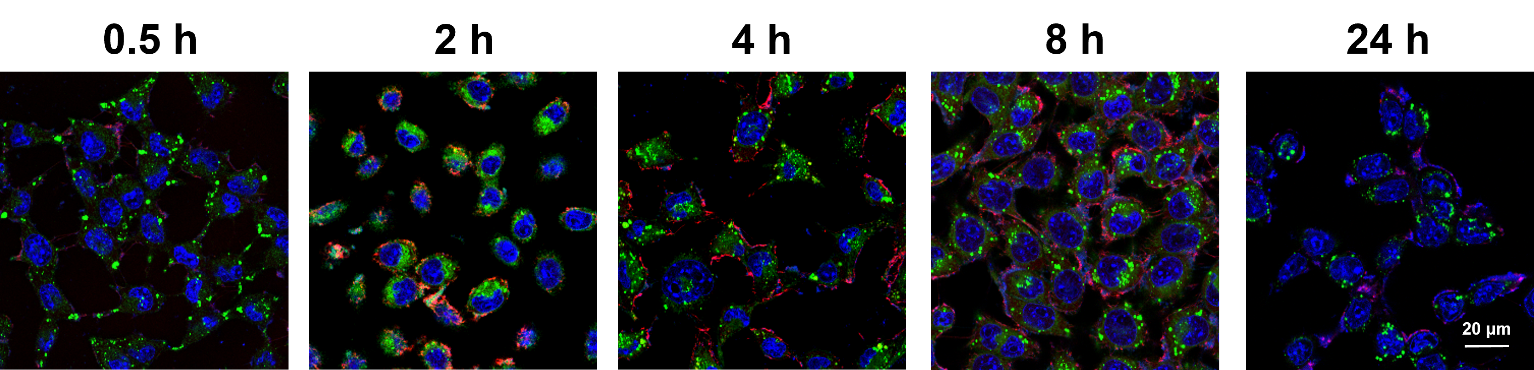


**Figure S19.** Time-dependent cellular internalization of SNPs visualized by CLSM.

**Figure S20.** Pearson’s correlation coefficient for colocalization of SNPs with lysosomes at different incubation times.


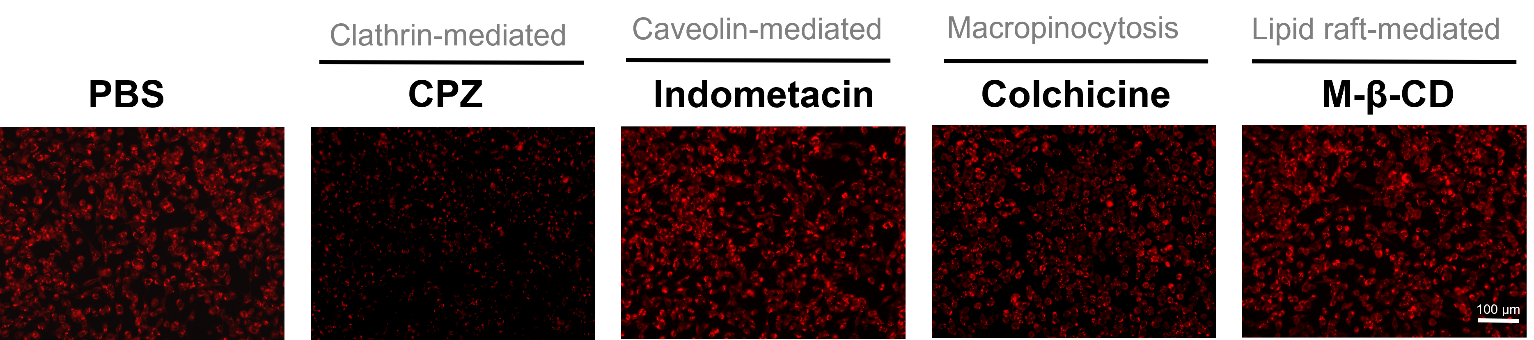


**Figure S21.** Effects of endocytosis pathway inhibitors on cellular uptake of SNPs.

**Figure S22.** Quantification of SNPs uptake in the presence of endocytosis inhibitors.


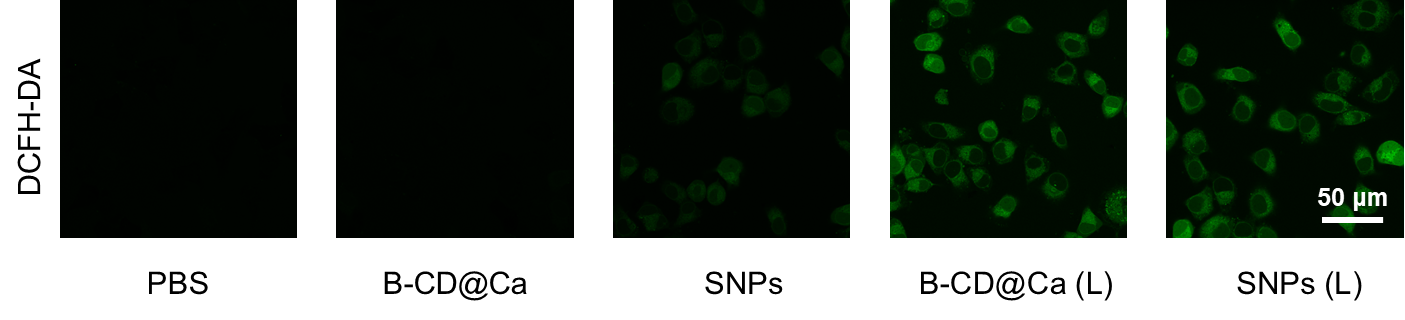


**Figure S23.** CLSM images of 4T1 cells stained with DCFH-DA probe.


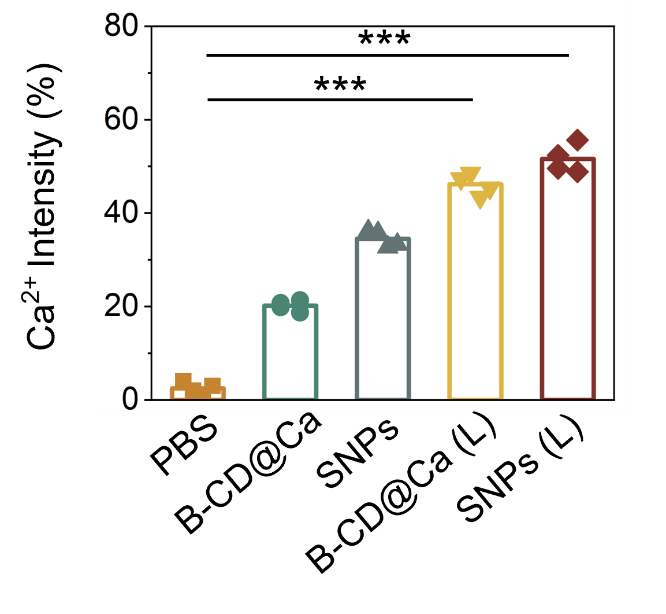


**Figure S24.** Quantification of intracellular Ca^2+^ levels.


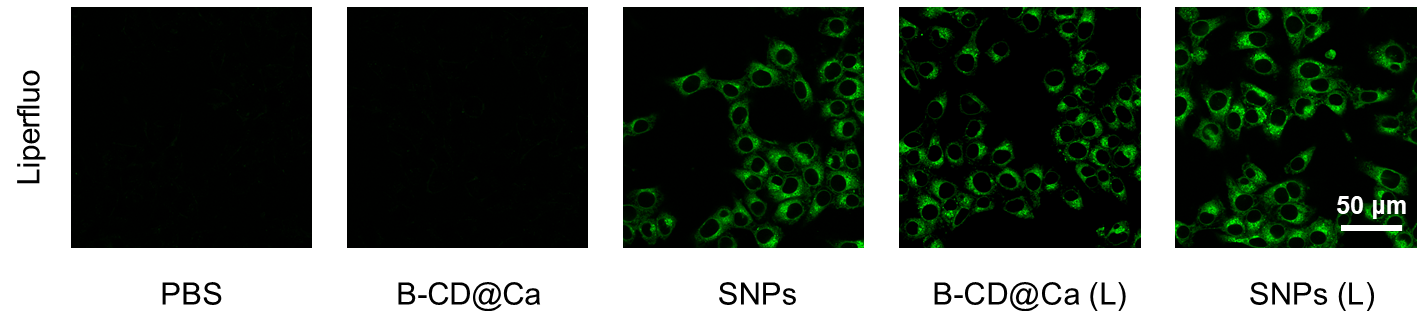


**Figure S25.** CLSM images of 4T1 cells stained with Liperfluo probe.


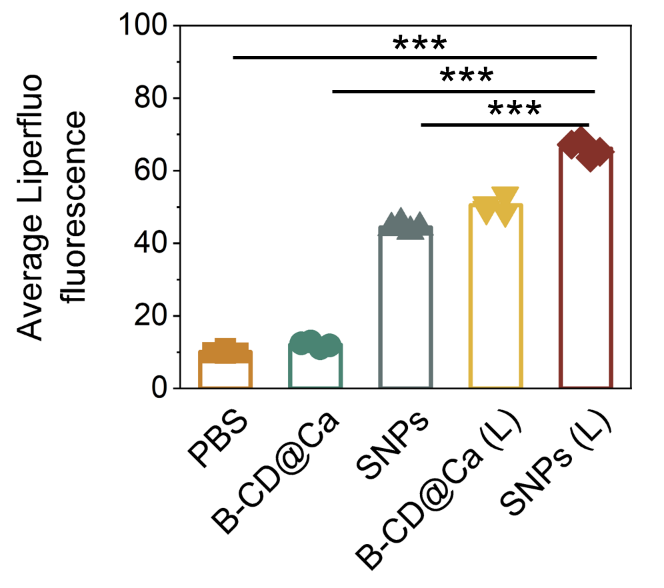


**Figure S26.** Quantification of LPO levels.


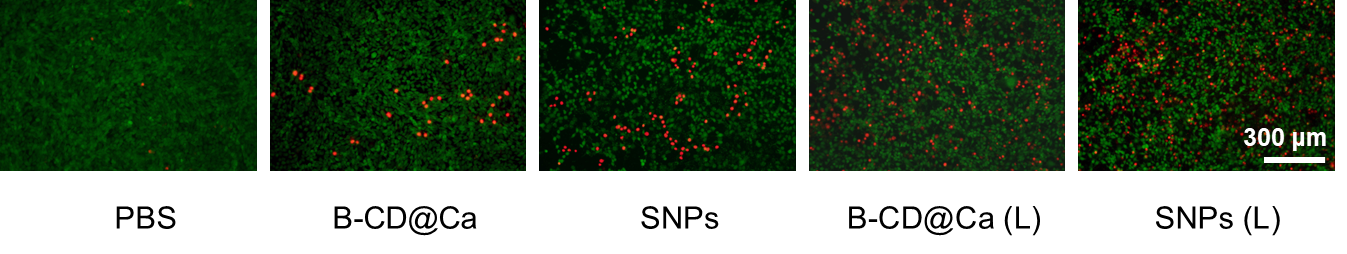


**Figure S27.** CLSM images of 4T1 cells stained with Calcein-AM and PI.


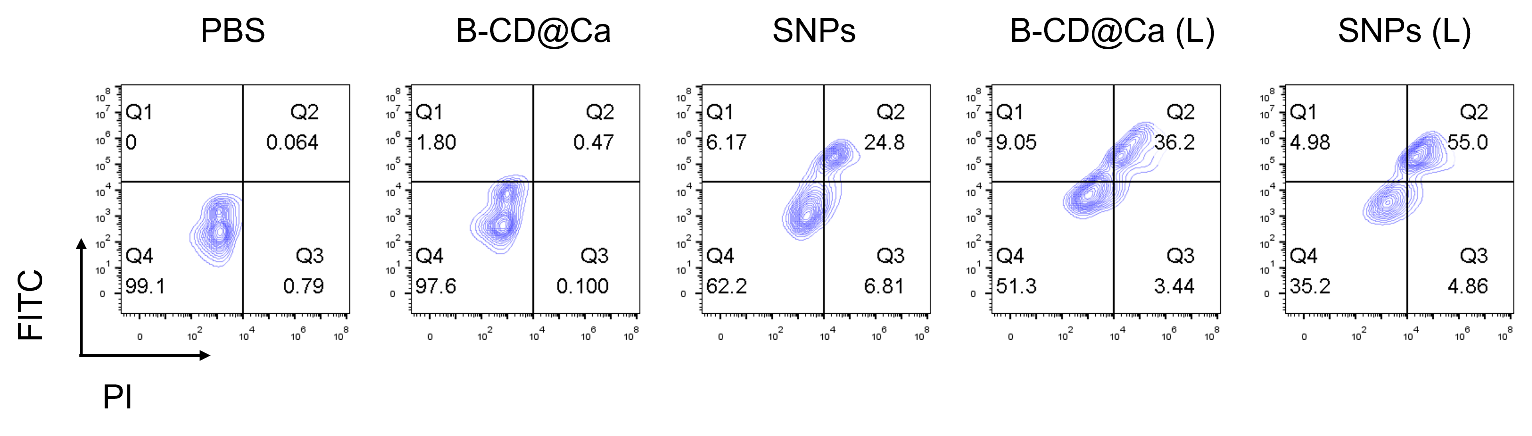


**Figure S28.** Flow cytometry analysis of 4T1 cells stained with Annexin V-FITC and PI.


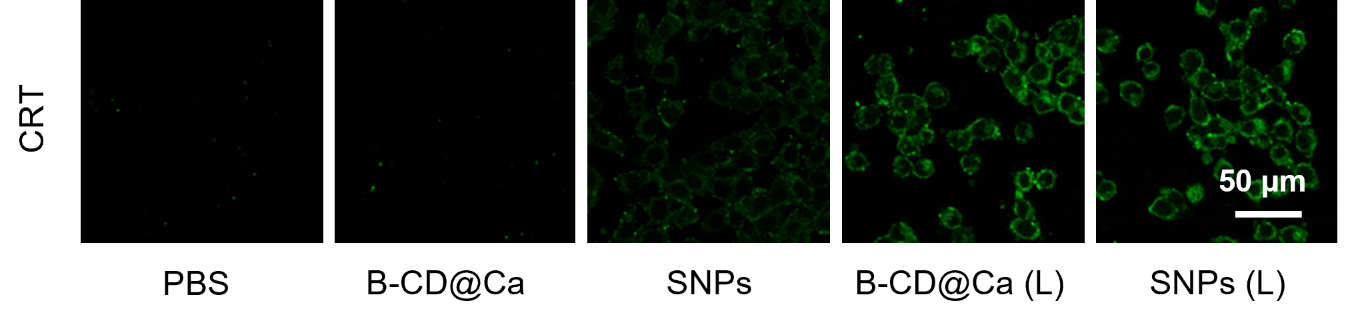


**Figure S29.** CLSM images showing CRT localization in 4T1 cells under different treatments.


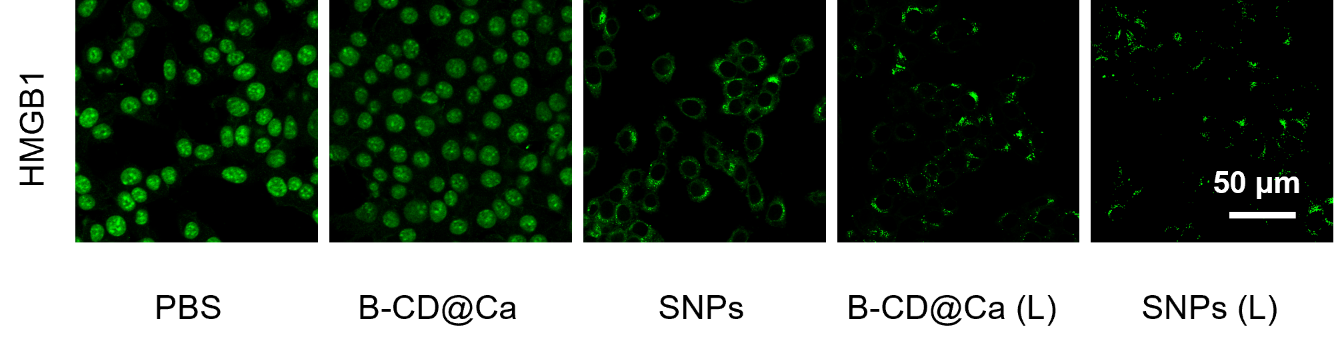


**Figure S30.** CLSM images showing HMGB1 localization in 4T1 cells under different treatments.


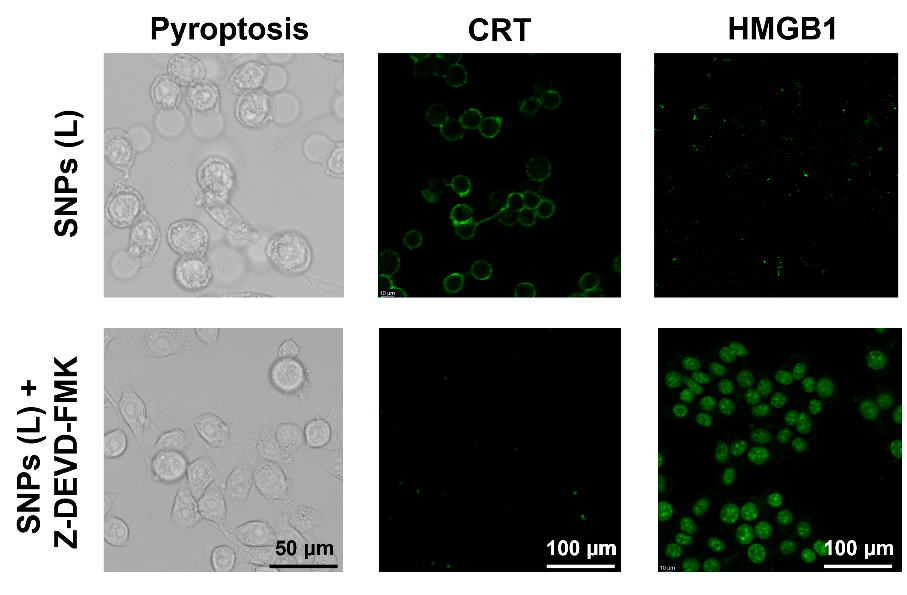


**Figure S31.** Effect of caspase-3 inhibitor on cell morphology, CRT exposure and HMGB1 translocation.


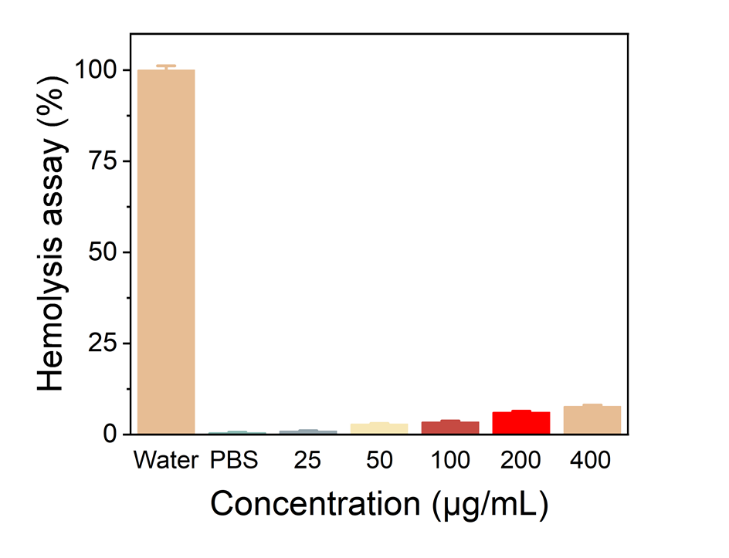


**Figure S32.** Hemolysis ratios of SNPs at various concentrations.


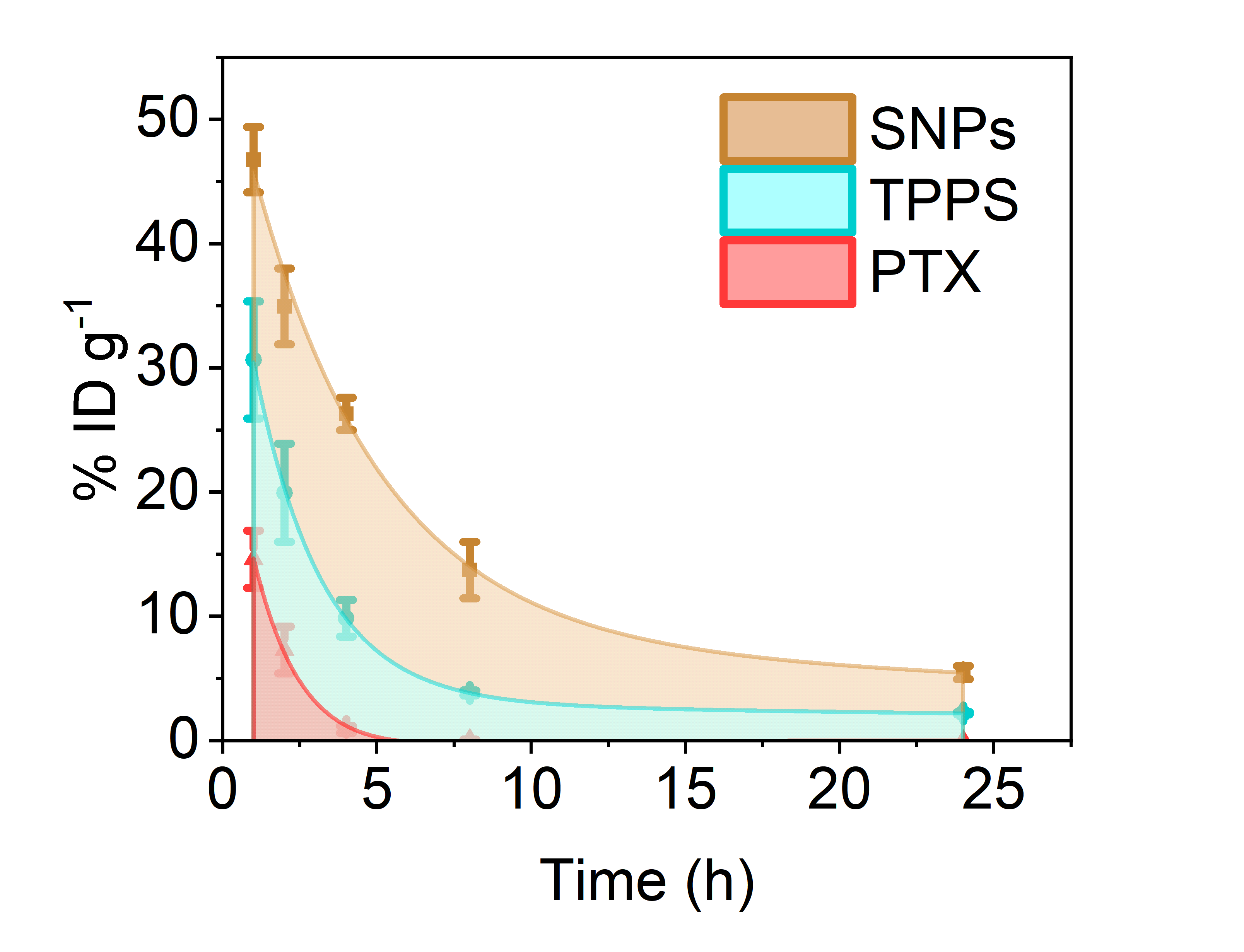


**Figure S33.** The pharmacokinetic profiles of TPPS, PTX and SNPs determined by HPLC and UV spectroscopy.


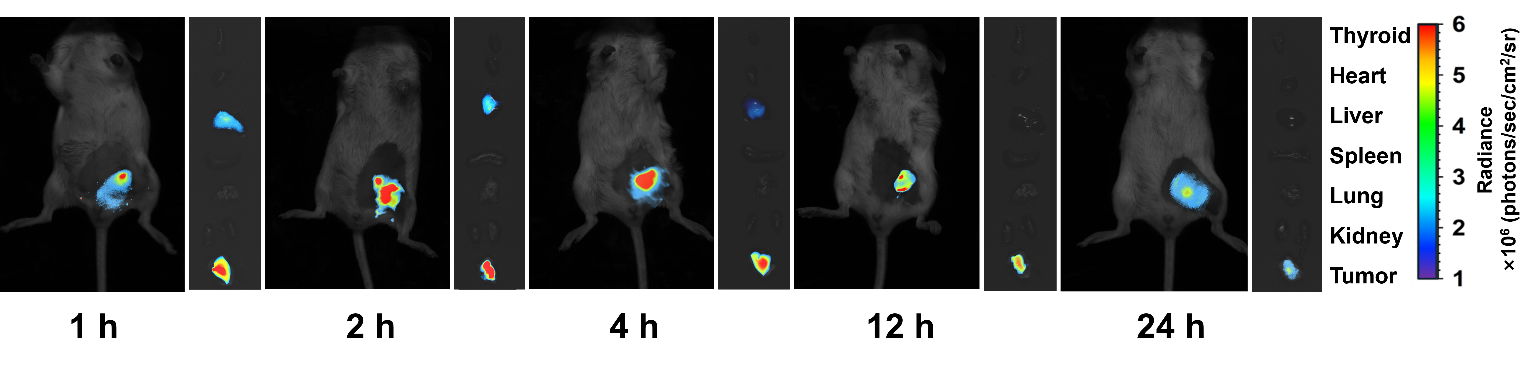


**Figure S34.** *In vivo* fluorescence whole-body imaging and corresponding *ex vivo* organ biodistribution at 1, 2, 4, 12 and 24 h post-injection.


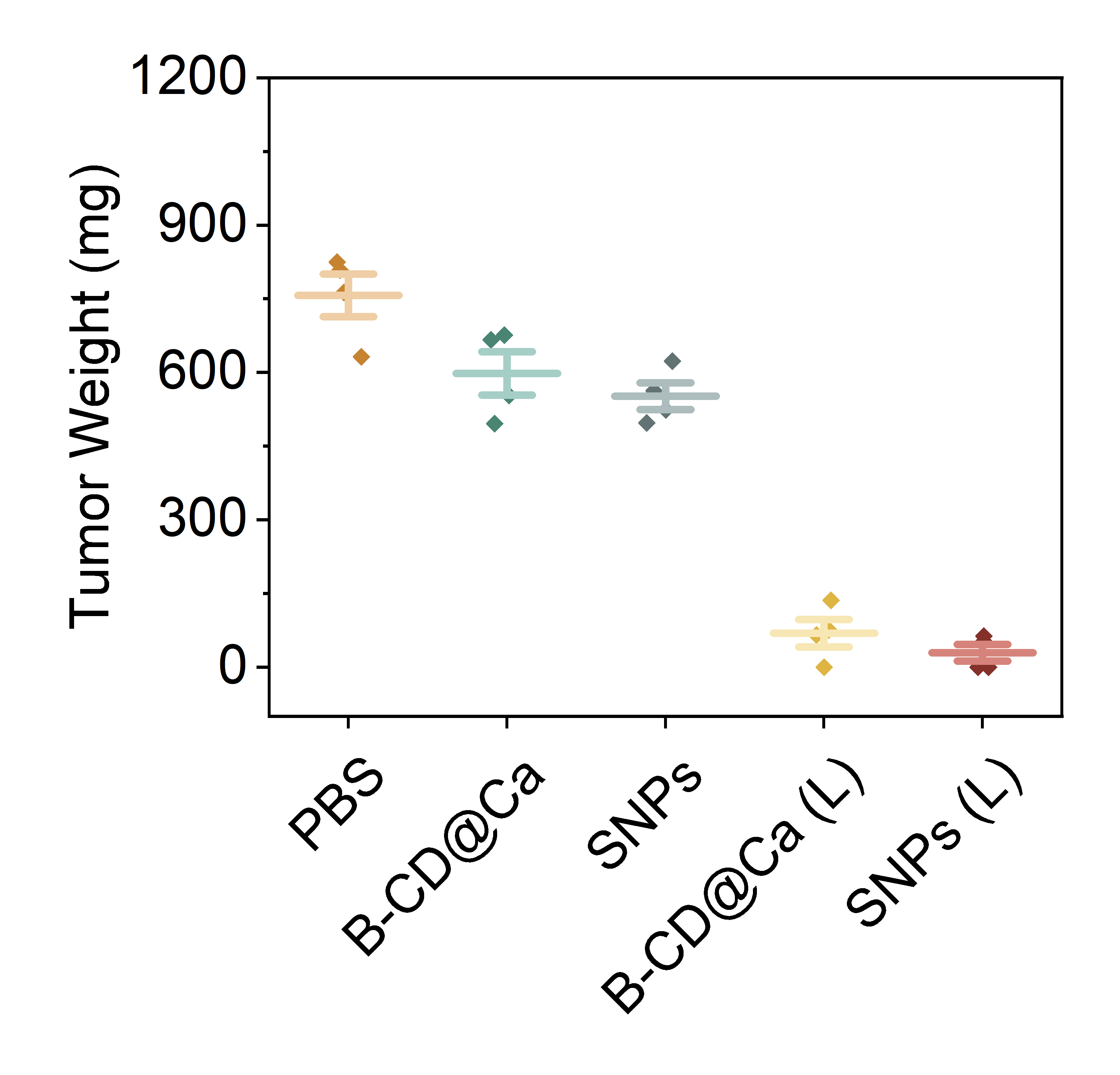


**Figure S35.** Average tumor weight of mice after different treatments.


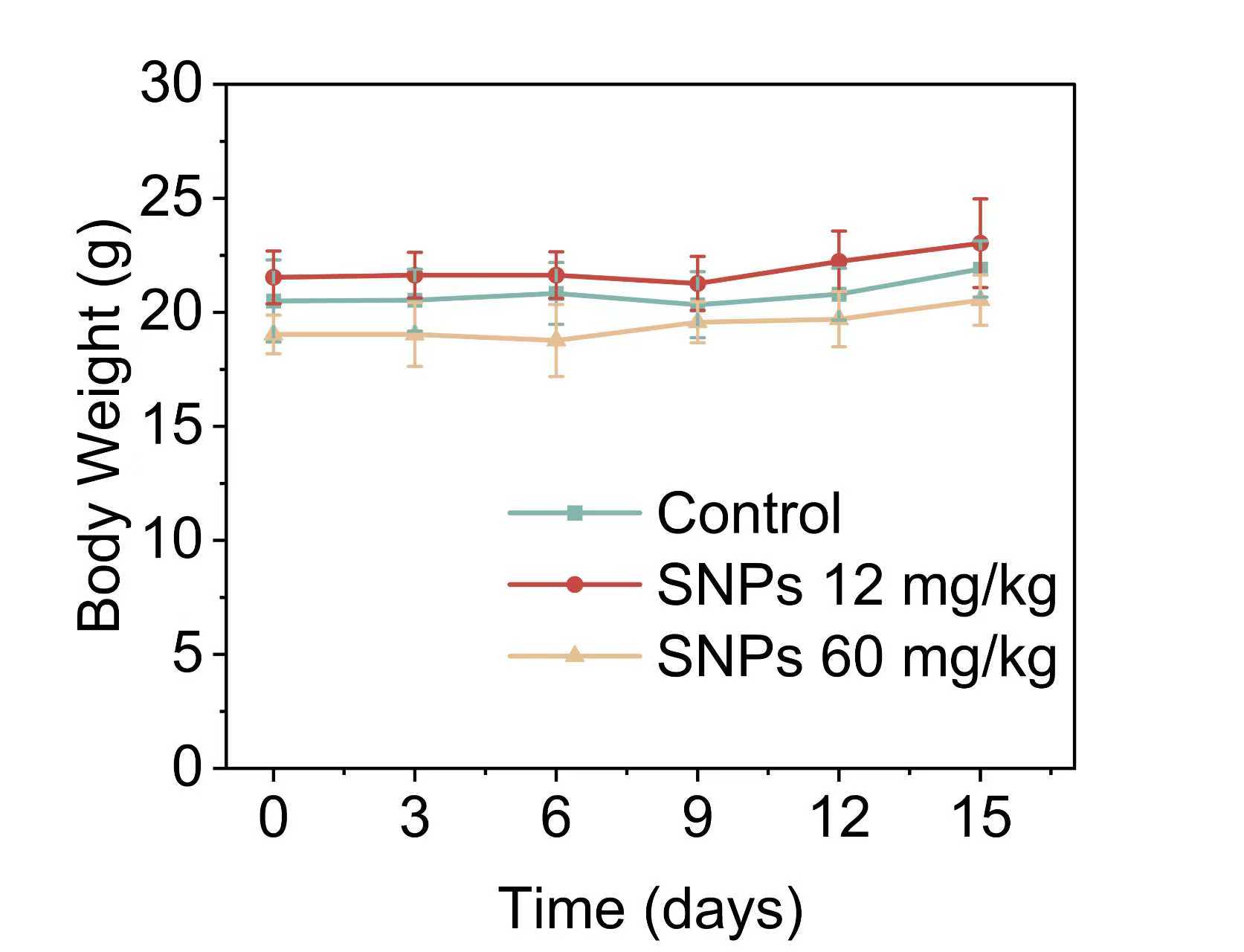


**Figure S36.** Body weight changes in mice during the acute toxicity assessment.


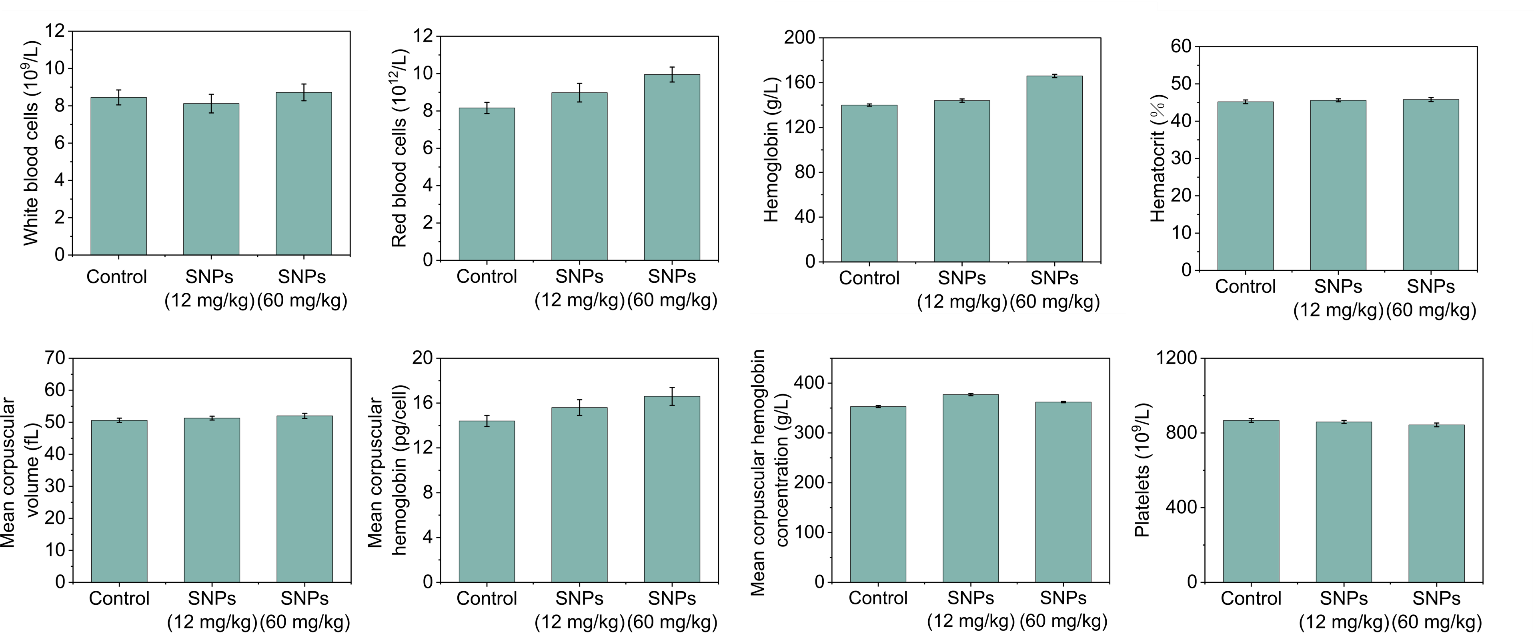


**Figure S37.** Hematological analysis of mice in an acute toxicity assessment.


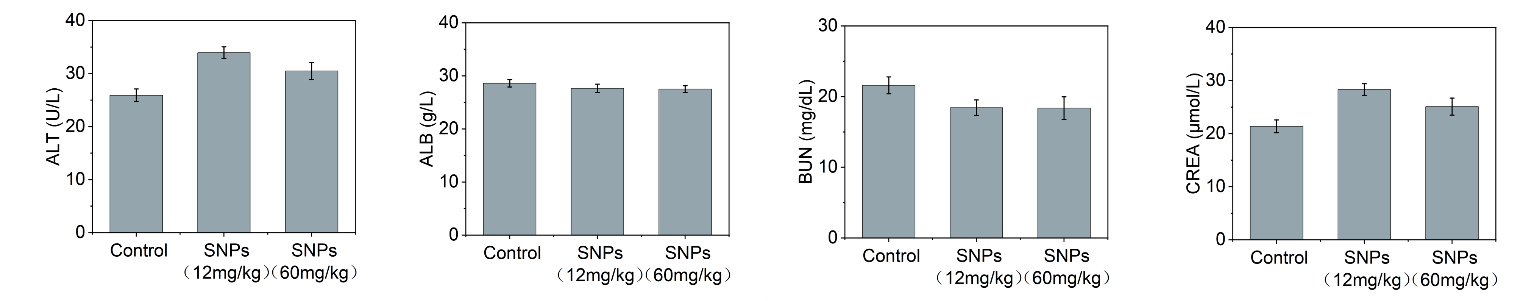


**Figure S38.** Assessment of liver and kidney function in mice following acute toxicity assessment.


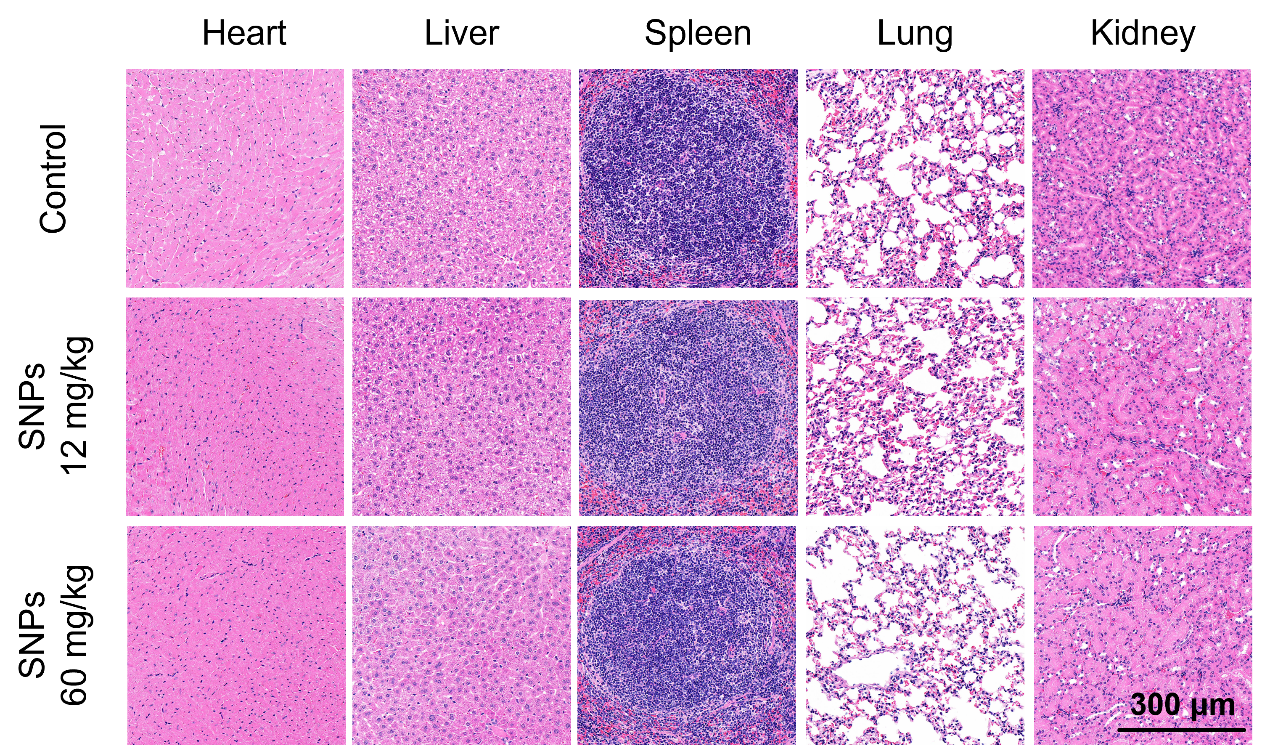


**Figure S39.** H&E staining of main organs from mice after acute toxicity assessment.


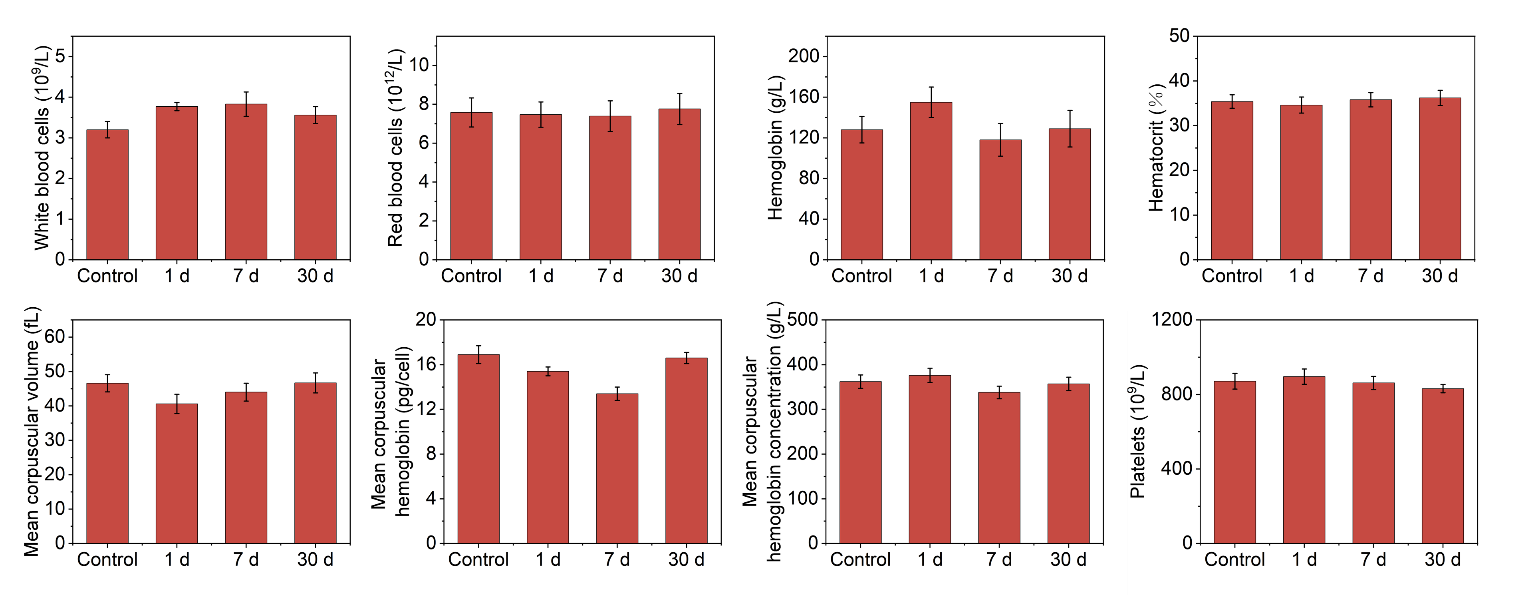


**Figure S40.** Hematological analysis of mice in a long-term toxicity assessment.


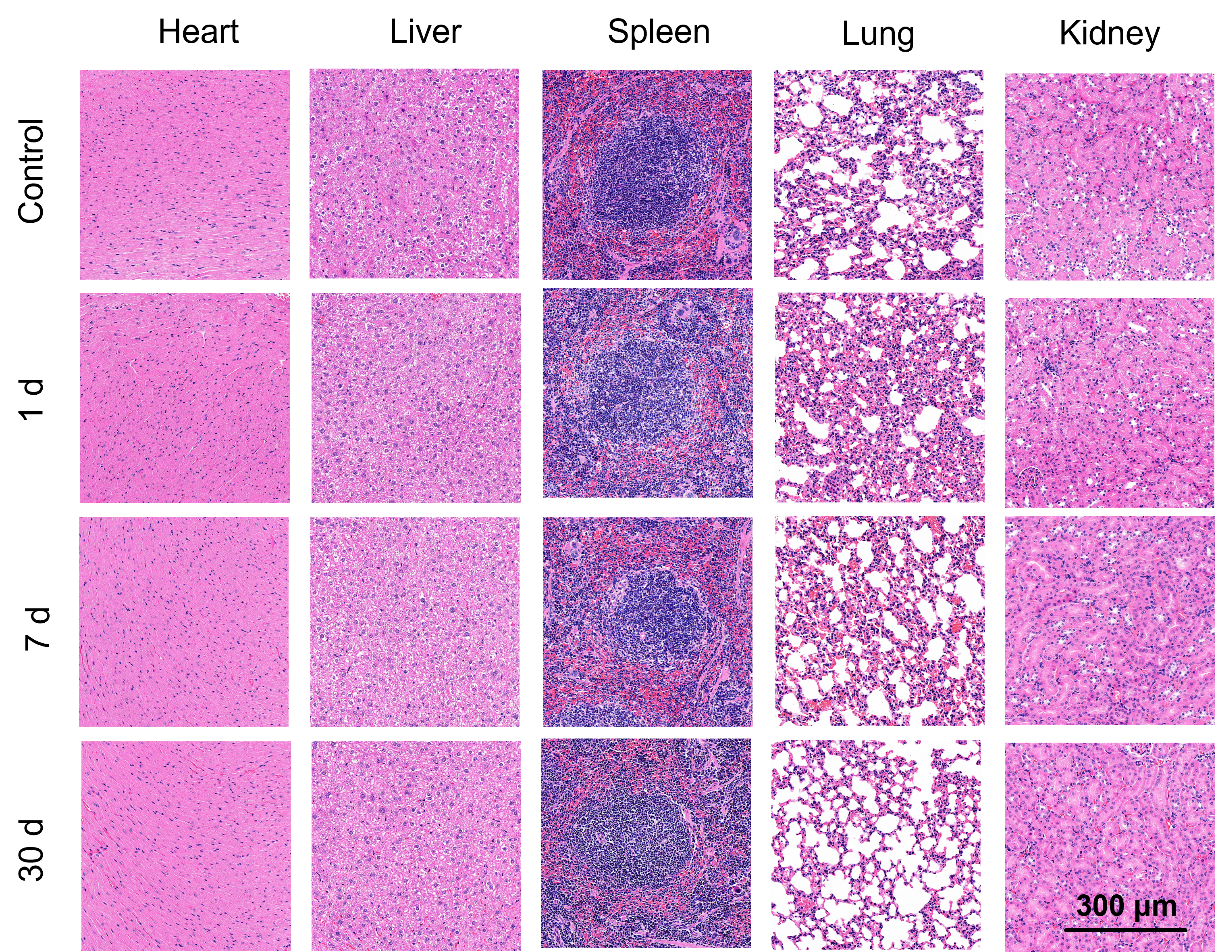


**Figure S41.** H&E staining of main organs from mice after long-term toxicity assessment.


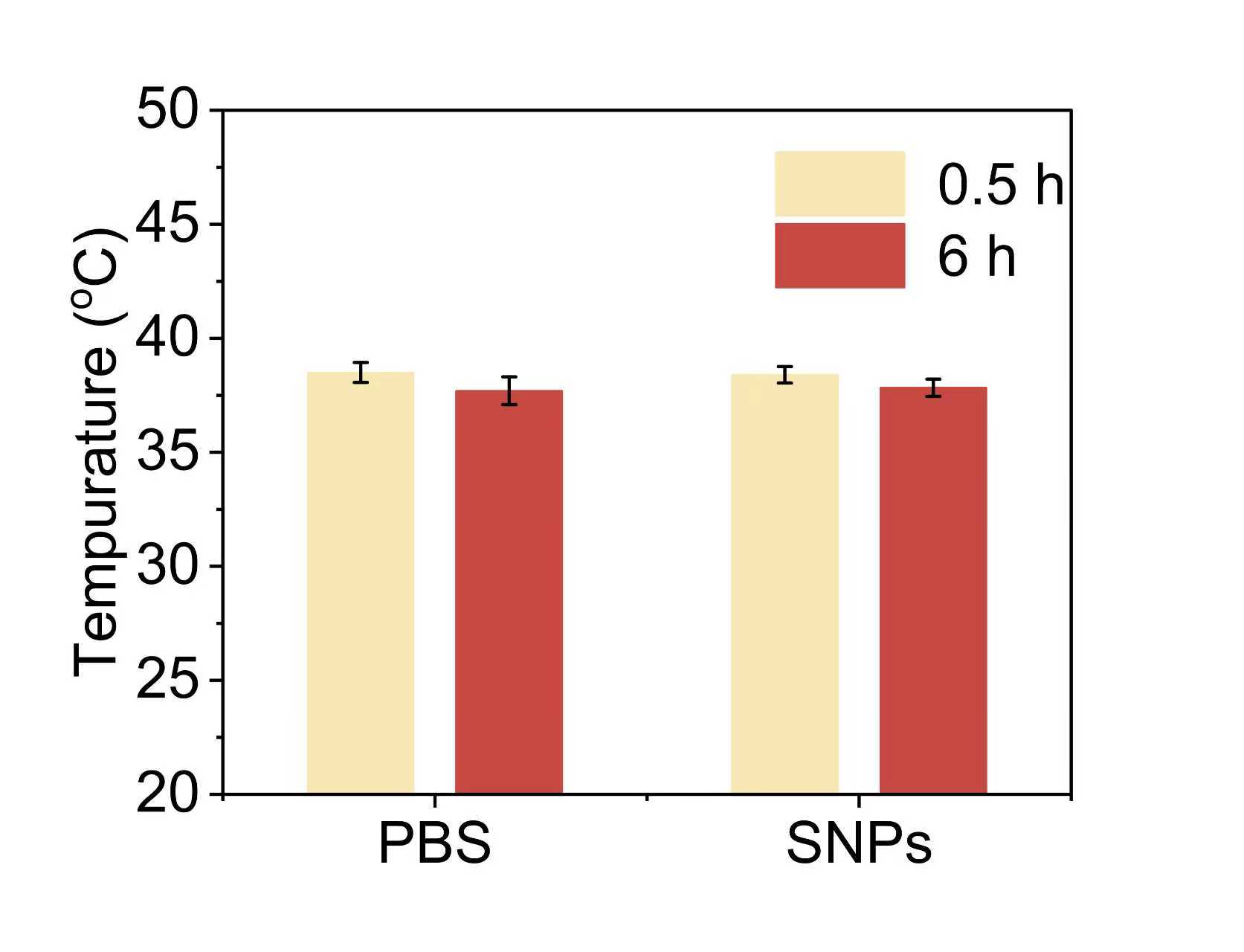


**Figure S42.** Body temperature changes during immunotoxicity assessment.


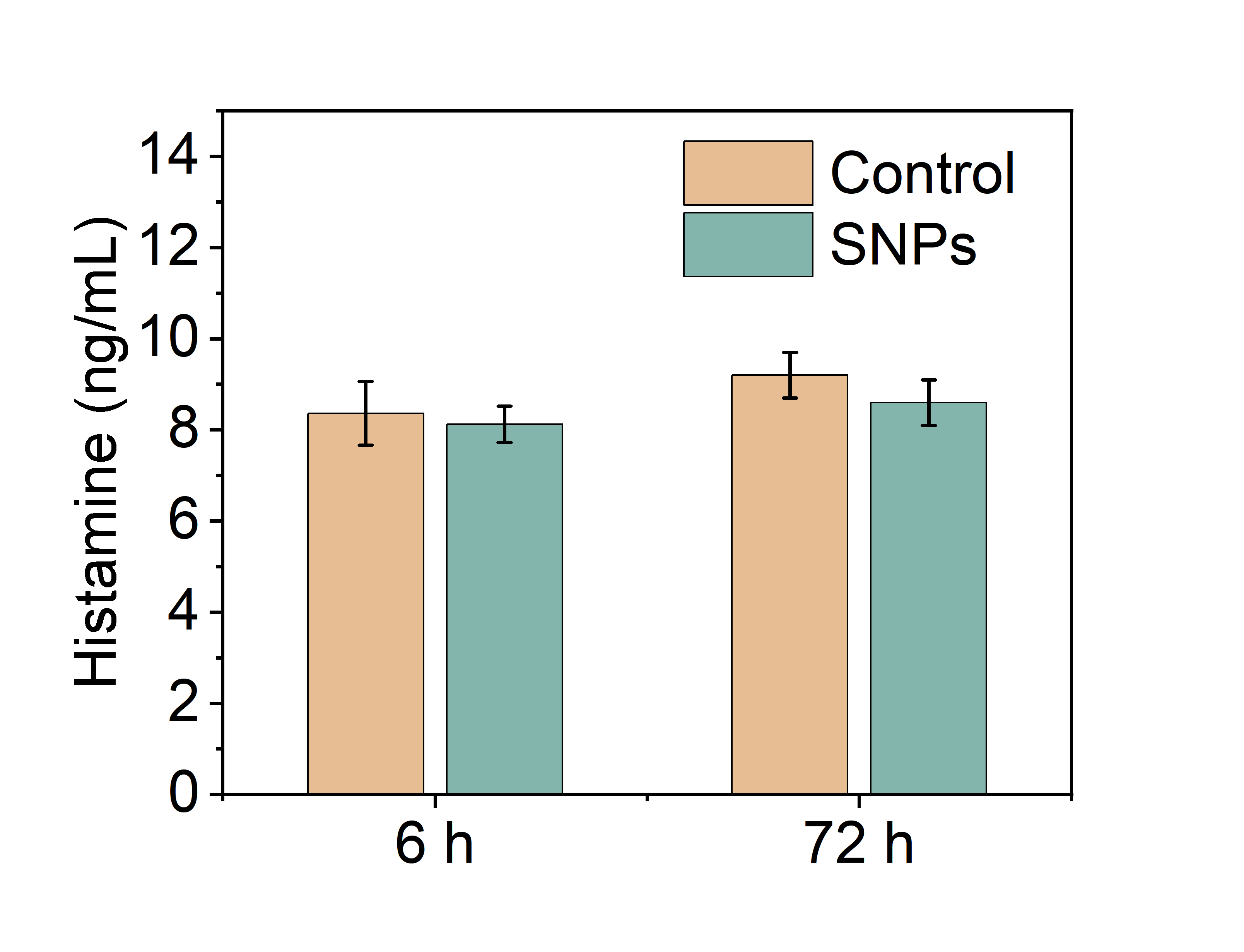


**Figure S43.** Serum histamine levels in mice during immunotoxicity assessment.


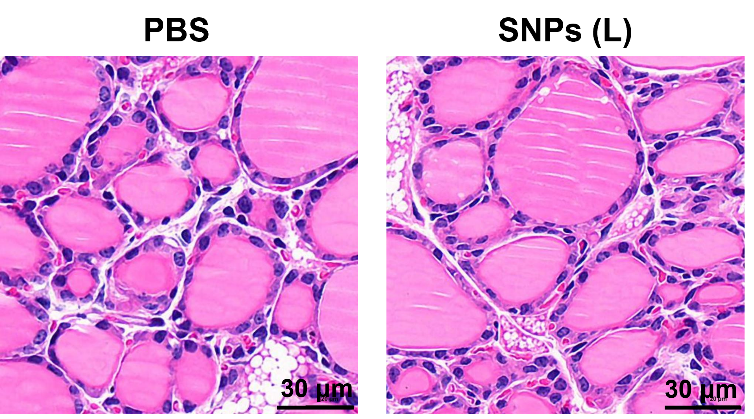


**Figure S44.** H& staining of thyroid tissues following different treatments.


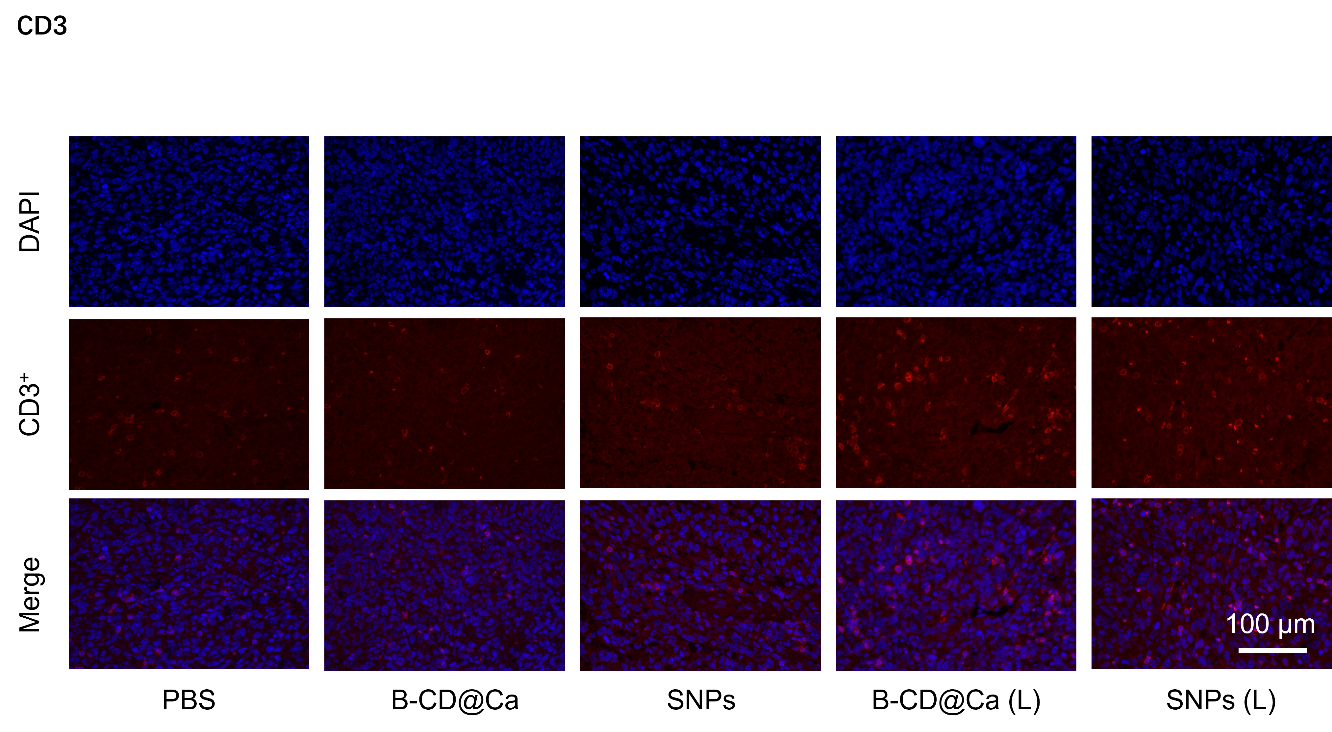


**Figure S45.** Immunofluorescence staining of CD3^+^ T cells of tumor tissues after different treatments.


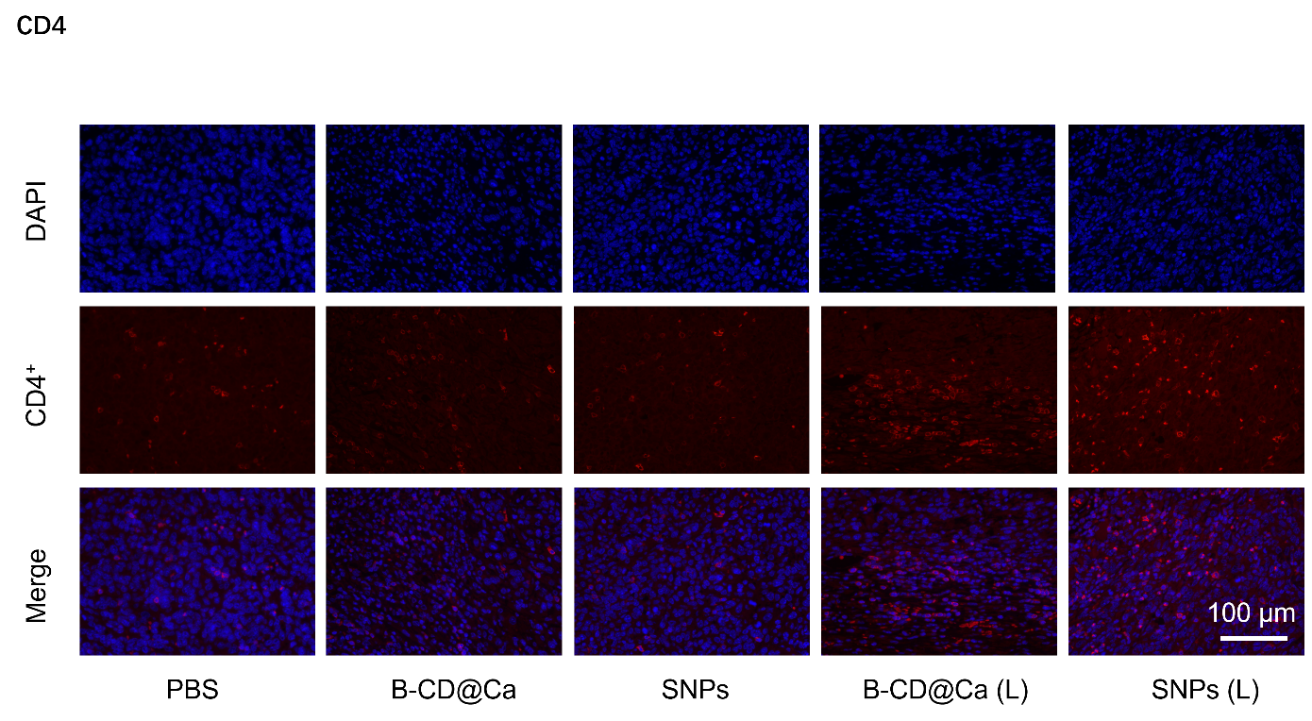


**Figure S46.** Immunofluorescence staining of CD4^+^ T cells of tumor tissues after different treatments.


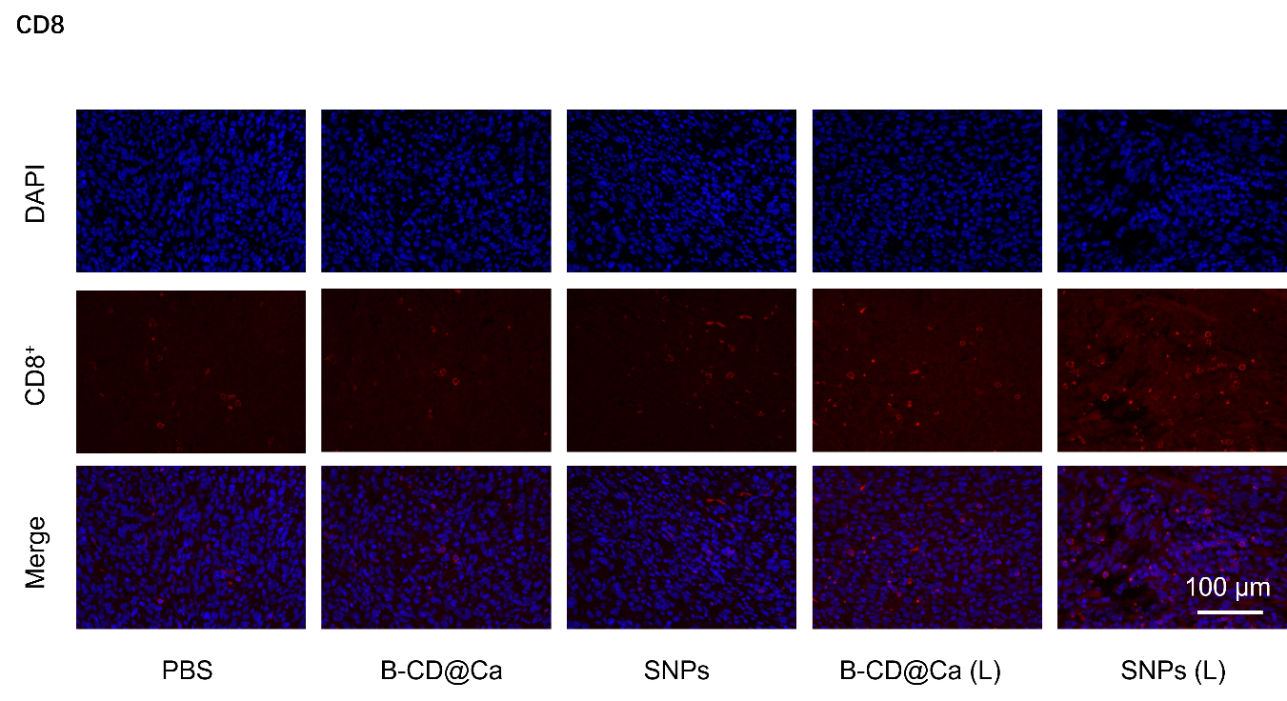


**Figure S47.** Immunofluorescence staining of CD8^+^ T cells of tumor tissues after different treatments.


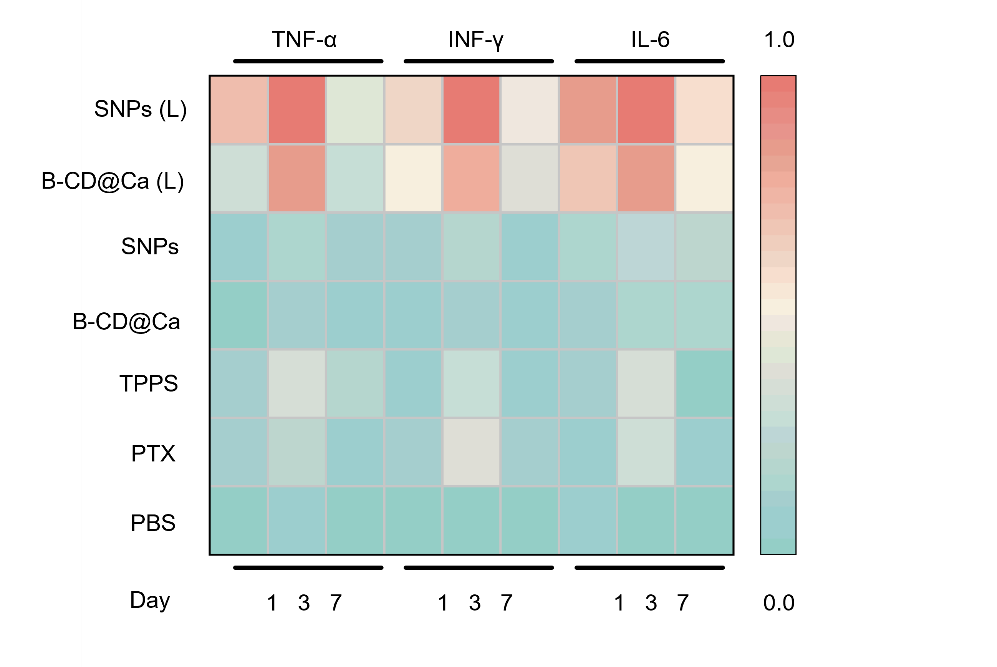


**Figure S48.** Serum levels of immunocompetent cytokines including IL-6, IFN-𝛾 and TNF-𝛼 following different treatments.


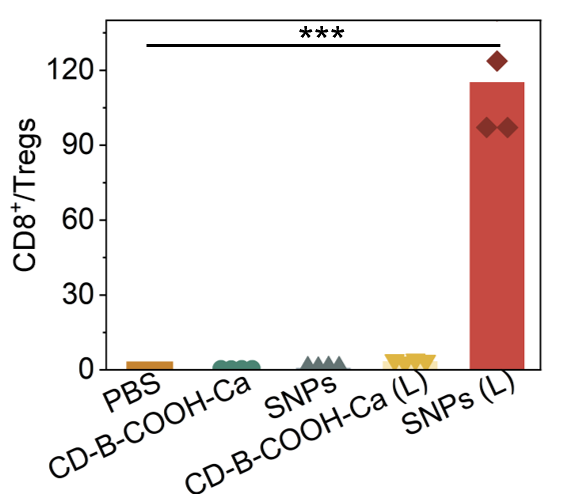


**Figure S49.** Flow cytometry analysis of CD8^+^/Tregs ratios.

**
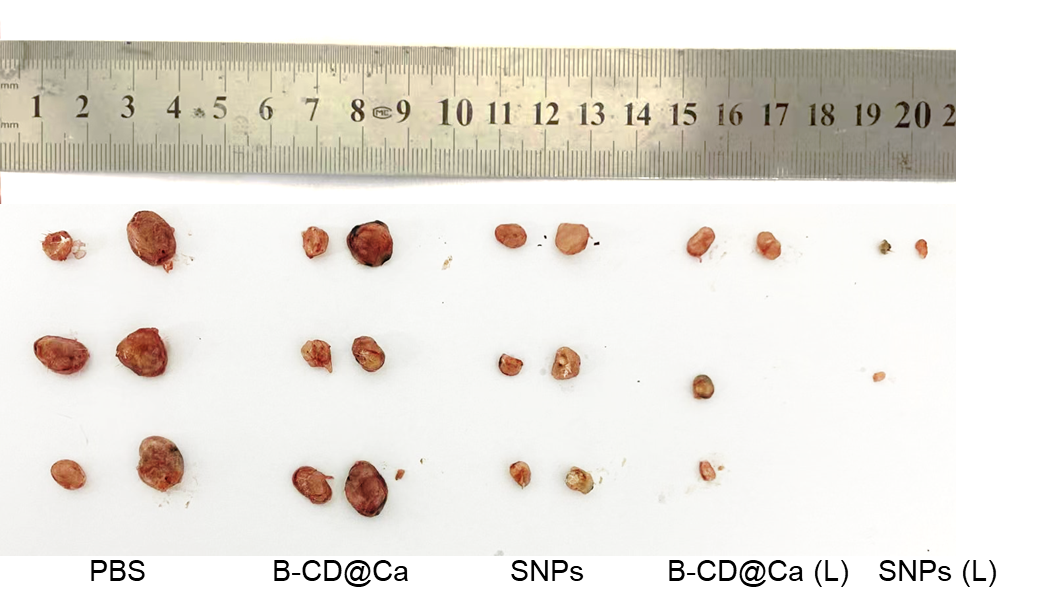
**

**Figure S50.** The picture of primary (left) and abscopal (right) tumor tissues after different treatments.


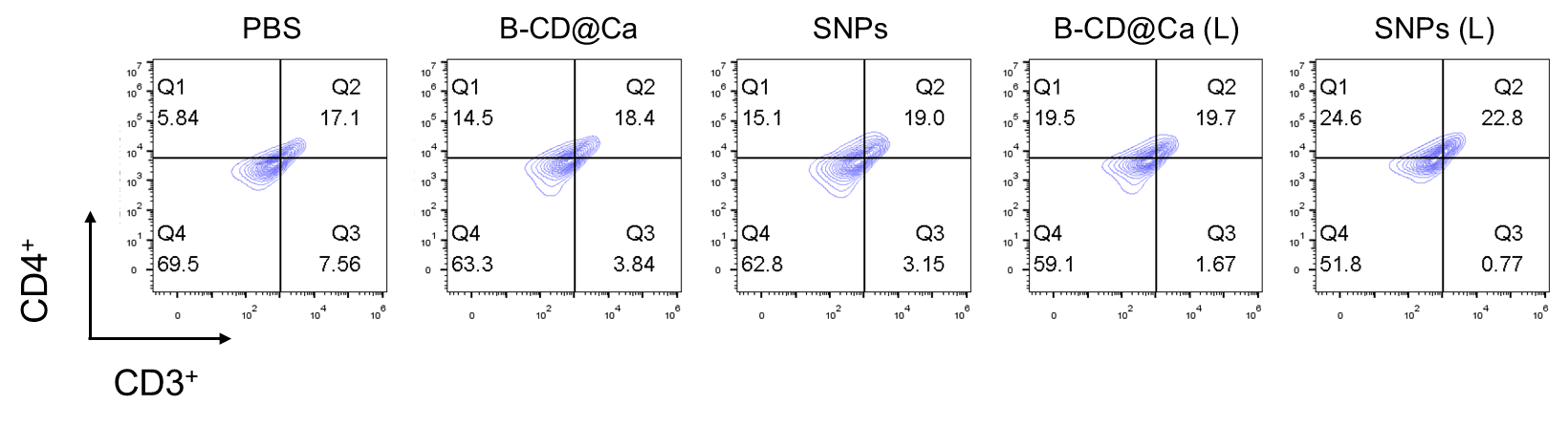


**Figure S51.** Flow cytometry analysis of CD3^+^CD4^+^ T cells in abscopal tumor tissues after different treatments.

**DPD simulations：**

The dissipative particle dynamics (DPD) method is a particle-based mesoscale simulation technique that includes explicit solvents. It was first introduced by Hoogerbrugge and Koelman^[3]^ in 1992 and improved by Español and Warren^[4]^. In DPD method, one bead represents a group of atoms. The bead’s internal degrees of freedom are integrated out and replaced by converse pairwise dissipative and random forces. Meanwhile, the motion of all the DPD beads obeys Newton’s equation of motion.

$$\frac{d\boldsymbol{r}_{i}}{dt}=\boldsymbol{\nu}_{i} , \frac{d\boldsymbol{\nu}_{i}}{dt}={\boldsymbol{f}_{i}}/{m_{i}} (1)$$

where ***r****_i_*, ***v****_i_* and m*_i_* denotes the position vector, velocity vector and mass of bead *i* respectively, and ***f****_i_* is the force acting on bead *i*.

**A. Interactions between DPD Beads.** In DPD method, the force ***F****_ij_* exerted on bead *i* by bead *j* is consisted of a conservative force $\boldsymbol{F}_{ij}^{C}$, a dissipative force $\boldsymbol{F}_{ij}^{D}$and a random force $\boldsymbol{F}_{ij}^{R}$. Thus, the total force ***f****_i_* is given by

$$\boldsymbol{f}_{i}=\sum_{j\neq i} \left( \boldsymbol{F}_{ij}^{C}+\boldsymbol{F}_{ij}^{D}\boldsymbol{+F}_{ij}^{R} \right) (2)$$

where the sum runs over all other beads within the cutoff radius *r_c_*. And the conservative force $\boldsymbol{F}_{ij}^{C}$, dissipative force $\boldsymbol{F}_{ij}^{D}$ and random force $\boldsymbol{F}_{ij}^{R}$ are given by

$$\boldsymbol{F}_{ij}^{C}=\left\{ \begin{aligned} a_{ij}\left( r_{c}-r_{ij} \right)\boldsymbol{e}_{ij} r_{ij}\leq r_{c} \\ 0 r_{ij}>r_{c} \end{aligned} \right. (3)$$

$$\boldsymbol{F}_{ij}^{D}= -\gamma\omega^{D}\left( r_{ij} \right)\left( \boldsymbol{e}_{ij}\cdot\boldsymbol{\nu}_{ij} \right)\boldsymbol{e}_{ij} (4)$$

$$\boldsymbol{F}_{ij}^{R}=\sigma\omega^{R}\left( r_{ij} \right)\theta_{ij}\boldsymbol{e}_{ij} (5)$$

where $a_{ij}$ is a constant that describes the maximum repulsion between two interacting beads.$r_{ij}$ is the distance between beads *i* and *j*. $\boldsymbol{e}_{ij}={\boldsymbol{r}_{ij}}/{r_{ij}}, \boldsymbol{\nu}_{ij}=\boldsymbol{\nu}_{i}-\boldsymbol{\nu}_{j},$ $\boldsymbol{\nu}_{i}\mathrm{and}\boldsymbol{\nu}_{j}$ are the velocities of beads *i* and *j*, respectively. *γ* and *σ* are the amplitudes of dissipative and random forces, respectively. $\theta_{ij}$ is a randomly fluctuating variable. *ω^D^* and *ω^R^* are r-dependent weight functions for dissipative and random forces, respectively. According to the fluctuation-dissipation theorem, *ω_ij_^D^*(*r*)=[*ω_ij_^R^*(*r*)]^2^ and *σ^2^*=2*γk_B_T* (*σ*=3 and γ=4.5)*.* The following simple form of *ω^D^* and *ω^R^* was chosen by Groot and Warren^[5]^:

$$\omega^{D}\left( r \right)=\left[ \omega^{R}\left( r \right) \right]^{2}=\left\{ \begin{aligned} \left( 1-r/{r_{c}} \right)^{2} r\leq r_{c} \\ 0 r>r_{c} \end{aligned} \right. (6)$$

Also, the chain beads of polymers are connected by a harmonic bond:

$$\boldsymbol{F}_{ij}^{B}=-\sum_{j} C^{B}\left( r_{ij}-r_{eq} \right)\boldsymbol{e}_{ij} (7)$$

where $C^{B} \mathrm{and}r_{eq}$ denotes the bond rigidity and the equilibrium bond length, respectively. Here, the most common and reliable values ($C^{B}=4.0$ and $r_{eq}=0$)[3-4] were adopted in our simulations.

**B. Model and Interaction Parameters.**

Considering to keep the volume of the coarse-grained particles substantially the same, the following coarse-graining scheme was used to construct simulation models. The simple coarse-grained models of the components used in this study are shown in Figure 2H.

The DPD interaction parameters (αij) can be estimated based on the relationship between α and the Flory-Huggins parameters χ established by Groot and Warren^[5]^,

$$\alpha_{ij}=\alpha_{ii}+3.27\chi_{ij}$$

where α_ii_=25 is for the same type of bead. In this paper, molecule B-CD-COOH is primarily composed of two main types of particles, where the hydrophilic part consists of 21 beads and the hydrophobic part consists of 6 beads (Figure 2H).

During the simulation, we defined the interaction parameters between particles based on their compatibility. For incompatible particles, the interaction parameter was set to 50, while for compatible beads, the parameter ranged from 26 to 30. Considering the strong electrostatic interactions between beads C and D, their interaction parameter was defined as 5. The interaction parameters between particles A1, A2 and C, D were set to 10. The detailed interaction parameters are listed in Table S1. Then, the DPD simulations were carried out in the NVT ensemble in a cubic box (40×40×40) under periodic boundary conditions. The system number density is set to 3. For simplicity, the cutoff radius *R_c_*, the bead mass *m*, and the temperature *k_B_T* are taken as the units of the simulations, i.e., *R_c_*=*m*=*k_B_T*=1; thus, the time unit *τ*= (*mR_c_*^2^/*k_B_T*)^1/2^=1. The GWVV algorithm was used to integrate motion equation [3] with ∆t = 0.04. During the simulation, the experimental procedure was divided into three steps: first, the self-assembly of molecule X1 in solution; followed by the addition of Ca²⁺ into the system; and finally, the introduction of CO₃²⁻ ions. Each step involved 1.5 million steps of DPD simulation. All the DPD simulations were performed by using Pygamd package^[6]^. Periodic boundary conditions were applied. The trajectory was visualized by OVITO Basic program (version 3.10.4)^[7]^.

**Table S1.** Repulsive parameters (*a_ij_*) between beads for DPD simulation used in this work.

|  | A1 | A2 | B | C | D | S |
| --- | --- | --- | --- | --- | --- | --- |
| A1 | 25.00 |  |  |  |  |  |
| A2 | 30.00 | 25.00 |  |  |  |  |
| B | 50.00 | 50.00 | 25.00 |  |  |  |
| C | 10.00 | 10.00 | 50.00 | 25.00 |  |  |
| D | 10.00 | 10.00 | 50.00 | 5.00 | 25.00 |  |
| S | 30.00 | 30.00 | 50.00 | 26.00 | 26.00 | 25.00 |

**References:**

1. Wu, D.; Zhou, J.; Zhang, Z.; Cao, Y.; Ping, K.; Qi, S.; Du, J.; Yu, G. Supramolecular Modulation of Tumor Microenvironment Through Host−Guest Recognition and Metal Coordination to Potentiate Cancer Chemoimmunotherapy. *Adv. Sci.* **2025**, *12*, e2408518.
2. Shen, J.; Wang, Q.; Lv, Y.; Dong, J.; Xuan, G.; Yang, J.; Wu, D.; Zhou, J.; Yu, G.; Tang, G.; Li, X.; Huang, F.; Chen, X. Nanomedicine Fabricated from A Boron-dipyrromethene (BODIPY)-Embedded Amphiphilic Copolymer for Photothermal-Enhanced Chemotherapy. *ACS Biomater. Sci. Eng.* **2019**, *5*, 4463−4473.
3. Hoogerbrugge, P. J.; Koelman, J. M. V. A. Simulating Microscopic Hydrodynamic Phenomena with Dissipative Particle Dynamics. *Europhys. Lett.* **1992**, *19*, 155−160.
4. Espanol, P.; Warren, P. Statistical Mechanics of Dissipative Particle Dynamics. *Europhys. Lett.* **1995**, *30*, 191−196.
5. Groot, R. D.; Warren, P. B. Dissipative Particle Dynamics: Bridging the Gap between Atomistic and Mesoscopic Simulation. *J. Chem. Phys.* **1997**, *107*, 4423−4435.
6. Zhu, Y.; Liu, H.; Li, Z.; Qian, H.; Milano, G.; Lu, Z. GALAMOST: GPU-Accelerated Large-Scale Molecular Simulation Toolkit. *J. Comput. Chem.* **2013**, *34*, 2197−2211.
7. Stukowski, A. Visualization and Analysis of Atomistic Simulation Data with OVITO-the Open Visualization Tool. *Modell. Simul. Mater. Sci. Eng.* **2010**, *18*, 015012.
